# HPLC-based quantification of bacterial housekeeping nucleotides and alarmone messengers ppGpp and pppGpp

**DOI:** 10.1038/s41598-017-10988-6

**Published:** 2017-09-08

**Authors:** Vallo Varik, Sofia Raquel Alves Oliveira, Vasili Hauryliuk, Tanel Tenson

**Affiliations:** 1 University of Tartu, Institute of Technology, Nooruse 1, 50411 Tartu, Estonia; 2 Department of Molecular Biology, Umeå University, Building 6K, 6L University Hospital Area, SE-901 87 Umeå, Sweden; 3 Laboratory for Molecular Infection Medicine Sweden (MIMS), Umeå University, Building 6K and 6L, University Hospital Area, SE-901 87 Umeå, Sweden

## Abstract

Here we describe an HPLC-based method to quantify bacterial housekeeping nucleotides and the signaling messengers ppGpp and pppGpp. We have replicated and tested several previously reported HPLC-based approaches and assembled a method that can process 50 samples in three days, thus making kinetically resolved experiments feasible. The method combines cell harvesting by rapid filtration, followed by acid extraction, freeze-drying with chromatographic separation. We use a combination of C18 IPRP-HPLC (GMP unresolved and co-migrating with IMP; GDP and GTP; AMP, ADP and ATP; CTP; UTP) and SAX-HPLC in isocratic mode (ppGpp and pppGpp) with UV detection. The approach is applicable to bacteria without the requirement of metabolic labelling with 32P-labelled radioactive precursors. We applied our method to quantify nucleotide pools in *Escherichia coli* BW25113 K12-strain both throughout the growth curve and during acute stringent response induced by mupirocin. While ppGpp and pppGpp levels vary drastically (40- and ≥8-fold, respectively) these changes are decoupled from the quotients of the housekeeping pool and guanosine and adenosine housekeeping nucleotides: NTP/NDP/NMP ratio remains stable at 6/1/0.3 during both normal batch culture growth and upon acute amino acid starvation.

## Introduction

Both the concentration and fractional distribution of nucleotide species are key indicators of the metabolic state within bacterial cells. Nucleotides perform various ‘household’ functions in the cell such as energy storage and serving as building blocks for macromolecules. In addition, bacteria possess an array of nucleotides that are not directly involved in metabolism, but rather serve as regulatory secondary messengers, such as cyclic AMP (cAMP), cyclic diguanylate (c-di-GMP) as well as guanosine pentaphosphate (pppGpp) and tetraphosphate (ppGpp), collectively referred as (p)ppGpp^1^. (p)ppGpp is a pleiotropic effector which at low concentrations during unperturbed growth fine-tunes bacterial physiology and growth rate, while its acute accumulation in response to various stress stimuli orchestrates the survival and virulence program, the so-called ‘stringent response’^2^. Production of (p)ppGpp affects the balance of nucleotides in two ways: via consumption of GDP/GTP and ATP during (p)ppGpp synthesis and by direct inhibition of both the guanylate^3^ and adenylate^4^ synthesis pathways. The change in nucleoside triphosphate (NTP) levels serves as a regulatory parameter in itself. In *Bacillus subtilis* and other Firmicutes, the concentration of NTP nucleotides act as a key regulator of transcription and directly affects RNA polymerase by changing both the balance of initiating nucleotides^5^ and via binding to the transcriptional repressor CodY, a direct regulator of more than 300 genes^6^. Another key physiological parameter is ‘adenylate energy charge’, or AEC, which is defined as a ratio between the concentrations of AMP, ADP, and ATP: [(ATP) + 1/2 (ADP)]/[(ATP) + (ADP) + (AMP)]^7^. AEC ranges from 1 to 0 and describes the saturation level of adenosine species with high-energy phosphate bonds. During steady state growth AEC is universally maintained between 0.8-0.95 in a wide variety of organisms, including bacteria, yeast, and mammalian cells^7–11^. A prolonged decrease in AEC to values below 0.5 is accompanied by loss of viability in *E. coli* cultures^9, 12^.

Quantification of nucleotides is technically challenging for several reasons. Firstly, the cellular turnover of certain nucleotide species is very rapid. ATP is extremely labile, with a half-life of around one-tenth of a second^13, 14^. While ppGpp is relatively stable, with a half-life that is estimated to range between 200 to 30 seconds^15–17^, pppGpp is turned over with a half-life of around 10 seconds^15^. Secondly, nucleotides break down during sample processing either due to enzymatic activity^18^ or due to the intrinsic chemical instability of specific nucleotides – e.g. (p)ppGpp is unstable under both alkaline and acidic conditions, and at elevated temperatures^19^. The third challenge is the sheer complexity of the cellular nucleotide pools that must be unambiguously resolved, identified, and quantified.

**Figure 1:**
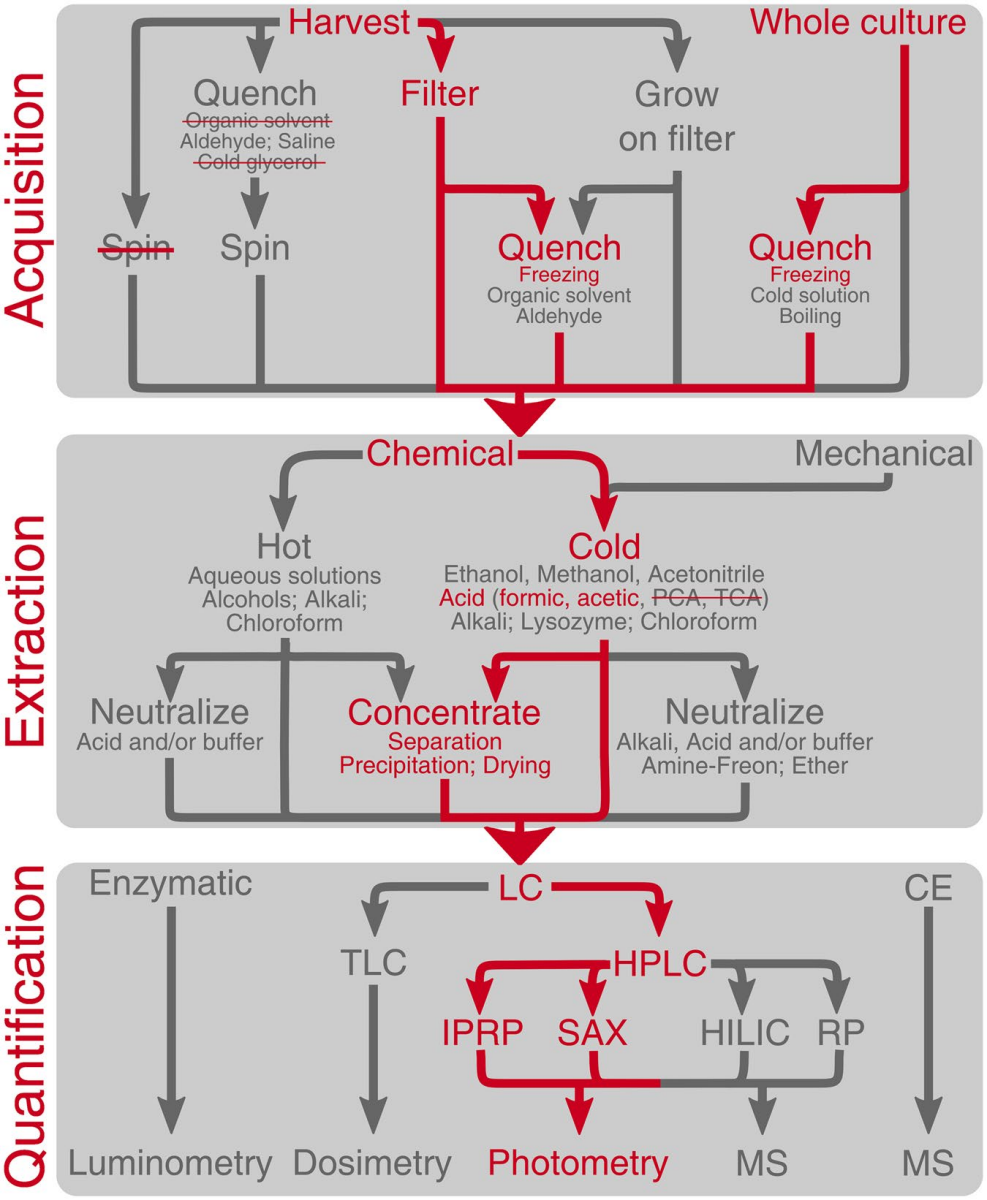
Generalized workflow for nucleotide quantification. The workflow can be subdivided into three steps: sample acquisition, nucleotide extraction, and quantification. Sample acquisition can be accomplished using either cell harvesting or whole culture sampling. The latter approach is less invasive and therefore less likely to introduce experimental artefacts. Cell harvesting using rapid filtration or growing cells directly on the filter is suitable for nucleotide analysis, however, centrifugation is not, because it perturbs nucleotide levels^9, 18, 20–22^, even when combined with quenching (Supplementary Figure 5a). During extraction, the nucleotide content is released from the cells either chemically or mechanically. When opting for cold acid extraction, one must consider the nature of the acid used. Some form of sample enrichment is often required when employing a whole culture approach. Finally, nucleotides are often quantified using liquid chromatography. Red arrows follow the approaches we have tested in the current report and recommend. Red strikethrough represents approaches that we urge one to avoid. PCA: perchloric acid; TCA: trichloroacetic acid; LC: liquid chromatography; TLC: thin layer liquid chromatography; HPLC: high pressure liquid chromatography; RP: reverse phase; IPRP; ion-paired reverse phase; SAX: strong anion exchange; HILIC: hydrophilic interaction chromatography; CE: capillary electrophoresis; and MS: mass spectrometry.

*Ex situ* nucleotide measurements can be divided into three steps: sample acquisition, extraction, and quantification (Fig. 1). Various experimental implementations of these three steps – as well as numerous pitfalls – are described in detail in the *Supplementary Materials*. The pitfalls are numerous and varied. For example, sample acquisition by centrifugation dramatically alters the nucleotide levels^9, 18, 20–22^; perchloric acid and trichloroacetic acid, TCA, are unsuitable for (p)ppGpp extraction^23^; and trace amounts of perchloric acid left in the sample after extraction interfere with HPLC analysis^24^.

Here we describe a method for nucleotide quantification that complies with the following requirements. First, it is capable of simultaneously following adenosine (ATP, ADP, and AMP), guanosine (GMP, GDP, GTP, ppGpp, and pppGpp) and pyrimidine (UTP, CTP) species. Second, it is applicable to bacteria growing in media which preclude the use of 32P-labelled radioactive precursors as quantification aids.Third, it is relatively high throughput and thus makes it feasible to conduct kinetically resolved experiments. We used this method to quantify the nucleotide pools in *Escherichia coli* BW25113 K12-strain both throughout the growth curve in minimal MOPS medium and during acute stringent response induced by mupirocin (pseudomonic acid), a competitive inhibitor of isoleucine aminoacyl-tRNA synthetase^25^.

## Results

### HPLC analysis of bacterial nucleotide mixtures by strong anion exchange chromatography, SAX

Interrogating the nucleotide content of complex biological materials using anion exchange chromatography dates back to at least the 1940s^26^. This approach matured by the 1980’s when 10 µm 4.6 × 250 mm SAX (Partisil) columns with irregular silica particles became the standard for nucleotide analysis^22, 27^.

**Figure 2:**
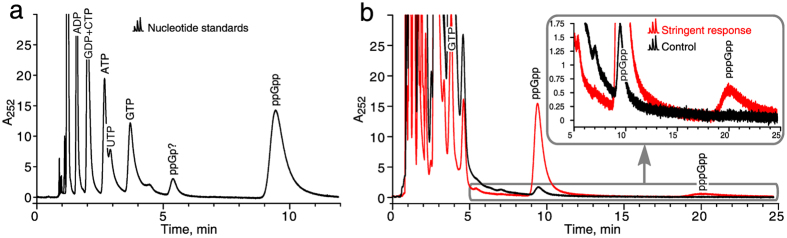
Isocratic strong anion exchange HPLC resolves ppGpp and pppGpp effectively, but is inefficient at resolving other nucleotide species. (**a**) Nucleotide standards (0.3 nmol each of ADP, GDP, CTP, ATP, UTP, GTP and 0.6 nmol of ppGpp) were run on isocratic SAX-HPLC using absorbance at 252 nm as a readout. (**b**) The system was used to resolve the nucleotides from *E. coli* cells both before and 5 minutes after induction of the stringent response. The stringent response was induced by mupirocin added to a final concentration of 150 µg/ml. Sample preparation is described in the Methods section. A 5 µm SphereClone column 4.6 $\times$ 150 mm was run with a buffer containing 0.36 M NH_4_H_2_PO_4_ pH 3.4, 2.5% acetonitrile at 26 °C at a flow rate of 1.5 ml/min.

We utilized a similar 5 µm 4.6 × 150 mm SAX column with spherical porous particles and employed either isocratic or gradient elution with ammonium phosphate buffers. Isocratic elution at pH 3.4 is suitable to quantify both ppGpp and pppGpp, but the resolution of other nucleotides – including GTP – is not robust (Fig. 2). Although the resolution of nucleotide standards is satisfactory under these conditions (Fig. 2a), separation of all major nucleotides extracted from the complex biological material in one isocratic run is not achievable (Fig. 2b).

**Figure 3:**
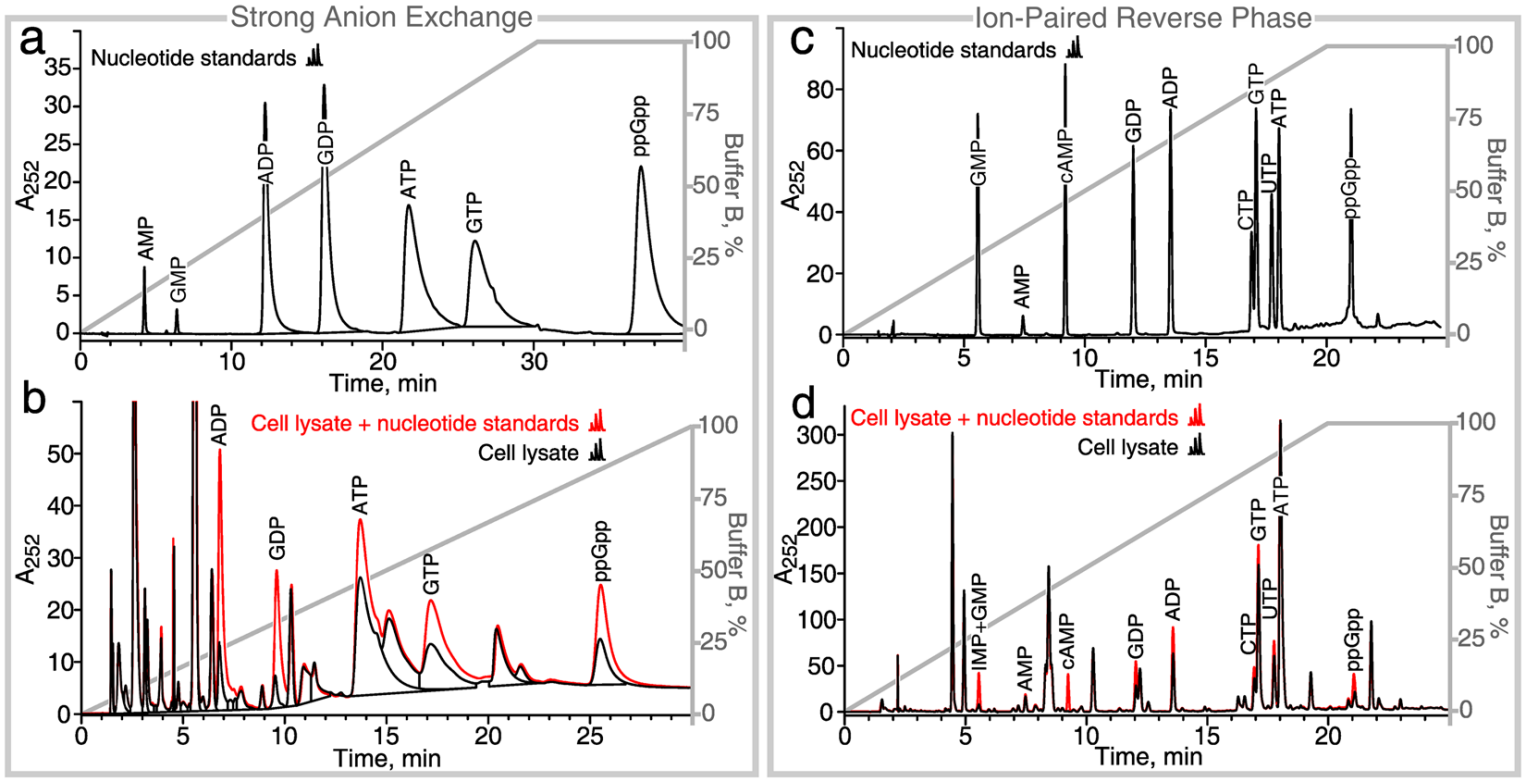
In gradient elution regime ion-paired reverse phase chromatography outperforms strong anion exchange HPLC both in sensitivity and resolution. (**a**) 2 nmol of nucleotide standard (ADP, GDP, ATP, GTP and ppGpp) were resolved in a SAX-HPLC run using gradient elution followed by tracking absorbance at 252 nm. Degradation of di-, tri- and tetraphosphates leads to the appearance of AMP and GMP in the standard. (**b**) Nucleotides extracted from an *E. coli* sample were resolved in a SAX-HPLC run using gradient elution both without (black trace) and with (red trace) a spiked-in 2 nmol nucleotide standard used to validate identity of the peaks. (**c**) 0.5 nmol of nucleotide standard (GMP, cAMP, GDP, ADP, CTP, GTP, UTP, ATP and ppGpp) were resolved in an IPRP-HPLC run using gradient elution. (**d**) Nucleotides extracted from an *E. coli* sample were resolved using IPRP with the aid of a spiked-in 0.25 nmol standard (red trace) used to validate the identity of the peaks. IMP and GMP were not resolved and co-migrate as one peak. Sample preparation is described in the Methods section. SAX-HPLC: A 5 µm Spherisorb 4.6 $\times$ 150 mm column was run at 1 ml/min and 26°C. Buffer A: 0.05 M NH_4_H_2_PO_4_, pH 3.4. Buffer B: 0.5 NH_4_H_2_PO_4_, pH 3.4. IPRP: Kinetix C18 2.6 µm 4.6 $\times$ 150 mm, 0.8 ml/min, 26°C. Buffer A: 5 mM Bu_4_NOH, 30 mM KH_2_PO_4_ pH 6.0. Buffer B: 100% acetonitrile.

While the use of gradient elution greatly improves the resolution of the nucleotide standard sample (Fig. 3a), it does not completely resolve the individual peaks of major nucleotide species when a considerably more complex mixture of nucleotides extracted from *E. coli* cultures is analyzed (Fig. 3b). Using a longer column does not increase the resolution due to significant widening of peaks (Supplementary Figure 1b). Moreover, aging of the SAX column leads to a significant decrease in retention times (compare retention times on Fig. 3a,b), which necessitates regular adjustments of the gradient and/or buffer strength. Despite these adjustments, quantification of ATP is not possible during the first 50 runs due to interfering peaks (see *Supplementary Methods*). To counter the aging effect, we strongly recommend re-calibrating the column by spiking nucleotide standards into cell lysate every 10–20 runs in order to validate the peak identities and adjust the run conditions across the lifespan of the column. A detailed protocol is provided in the *Supplementary Methods*.

### HPLC analysis of bacterial nucleotide mixtures by ion-paired reverse-phase chromatography, IPRP

Ion-paired reverse-phase chromatography (IPRP-HPLC) has been used for nucleotide analysis by several research groups^20, 22, 24, 28, 29^. Our implementation of this technique is based on the protocol by Payne and Ames who used a C18 column^22^. These columns are available in porous and improved pellicular form with shorter run times and increased sensitivity; the latter variant was used in the current study.

The IPRP approach has several advantages over SAX. First, the peaks are considerably narrower, are better resolved, and display more sensitivity (compare Fig. 3c,d: despite a four-fold lower amount of the standard, the peaks are still higher in the case of IPRP-HPLC). Despite the sharpness of the peaks, we recommend to use peak area – rather than height – to quantify IPRP-HPLC data. Second, the retention times are more stable throughout the column lifespan (compare Fig. 3c,d). Third, IPRP-HPLC does not require high salt buffers which can be detrimental to the HPLC instrument itself. Fourth, it is possible to resolve dNTP and NTP species (Supplementary Figure 3b,c; see also^30–32^).

In our hands, the typical lifespan of a pellicular C18 column is 250 runs when processing complex biological samples. However, after 30–50 runs the ppGpp peak starts to deform and splits into two overlapping areas: a sharp peak and a flat one (Supplementary Figure 2d). Implementation of the IPRP analysis by Payne and Ames^22^ also results in a similar shape of the ppGpp peak (see Fig. 2 in the original report). The most likely reason for the deformation of the (p)ppGpp peak is the accumulation of divalent metal ions in the column, because a wash step with 50 mM EDTA (pH 8.0) restores the initial shape of the peak (Supplementary Figure 2f). In addition, EDTA reduces the peak width, most notably the part closest to the baseline, and increases the peak height of triphosphate nucleotides. We have thus included this wash in our IPRP-HPLC routine (see *Supplementary Methods*).

Unfortunately, despite all of the above-mentioned measures, we failed to implement an IPRP-HPLC protocol that is capable of reliably quantifying (p)ppGpp using a UV detector. In unstressed *E. coli* cells, the level of (p)ppGpp is typically below the limit of detection (Supplementary Figure 4a,b). Upon acute amino acid starvation, ppGpp becomes readily detectable, although the resolution of the ppGpp peak is poor and the baseline around the ppGpp peak tends to elevate, which confounds the quantification of this nucleotide (Supplementary Figure 4a,b and 10a–d). Ultimately, we were not able to detect pppGpp even in stressed cells (Supplementary Figure 4c,d).

To conclude, while our IPRP method provides excellent resolution, speed and sensitivity for the quantification of most housekeeping nucleotides (GDP and GTP; AMP, ADP and ATP; CTP; UTP), it is poorly suited to quantify GMP (which co-elutes with IMP), ppGpp, and pppGpp. The latter two nucleotides can be readily quantified using SAX (see the previous section for details).

### Sample preparation without harvesting

We based our protocol on that of Buckstein and colleagues^20^. Their workflow consists of the following steps. The whole culture broth is sampled by pouring into cold formic acid followed by freezing in liquid nitrogen. After that, the mixture is carefully thawed and extraction continues on ice for half an hour. The next step aims to crudely purify and concentrate the nucleotides, as well as to remove the acid: the diluted acidic sample is loaded on a SAX-FPLC and eluted with 1 M ammonium formate buffer. After that the sample is desalted by dialysis in sucrose solution for 40 hours, the nucleotides are concentrated via freeze-drying, taken up in water and analyzed on HPLC.

We redesigned the entire workflow with the aim of increasing its throughput. Specifically, we removed the tedious and time-consuming dialysis step, which halves the sample processing time from four to two days. Following advice from Dr. Michael Cashel, we opted to use 2 M lithium chloride instead of 1 M ammonium formate during the FPLC elution step. Our final step is precipitating the mixture overnight in ethanol at −20 °C which refines the mixture by selective precipitation of nucleotides. To estimate the nucleotide loss during the entire course of the sample preparation procedure, we spiked in nucleotide standards – ADP, ATP, GDP, GTP, and ppGpp – into the frozen cell sample and calculated their recoveries (Table 1). The recoveries range from 92% (ppGpp) to 60% (GTP), with the exception of ADP – 17%. Neither AMP nor GMP could be detected within the cell lysate, and thus their recoveries were not estimated. A direct comparison of the recoveries of our approach with the original method by Buckstein and colleagues^20^ is problematic because, unlike us, Buckstein and colleagues report the nucleotide recoveries using pure standards alone rather than spiked into cell lysate.

**Table 1:** Nucleotide recoveries. Known amounts of nucleotide standards were added to snap frozen cells that were then processed and analyzed using either SAX (ppGpp) or IPRP (ADP, GDP, ATP and GTP). See the Methods section for details. The recovery was calculated by comparing increase in signal resulting from the addition of a nucleotide standard with the signal obtained using a pure standard (n ≥ 8). 95% CI: 95% confidence interval. *NA*: not available.

Nucleotide	Whole culture sampling	Filtration sampling
	mean_[95% CI]_, %	mean_[95% CI]_, %
AMP	*NA*	85_[80; 89]_
ADP	17_[15;19]_	86_[84; 88]_
ATP	75_[63; 90]_	82_[76; 89]_
GMP	*NA*	83_[80; 85]_
GDP	72_[68; 77]_	82_[77; 88]_
GTP	60_[50; 73]_	66_[63; 69]_
ppGpp	92_[83; 100]_	46_[40; 52]_
UTP	*NA*	92_[89;94]_
CTP	*NA*	80_[70; 93]_

The main limitations of our whole culture approach are its inability to retain monophosphates and the poor recovery of ADP. Although we were able to halve the sample processing time, it remains a labor-intensive and time-consuming procedure: Only 6 to 8 samples can be processed in three days. To overcome these limitations, we have applied sampling via harvesting.

### Sample preparation with a harvesting step

Harvesting results in more concentrated starting material and renders the enrichment step using FPLC and/or precipitation unnecessary. This shortens the procedure and counters the loss of monophosphates and ADP compared with the whole culture approach described above. While centrifugation is a convenient approach for collecting bacterial cells, several reports suggest that it significantly perturbs the nucleotide composition of bacteria^9, 18, 20–22^. Several groups have used an additional quenching step prior to centrifugation using cold glycerol^33^ or aldehyde fixation^34^. We tested whether quenching can mitigate the perturbation of nucleotide levels induced by centrifugation. To assess the performance of the method we quantified both the ATP/ADP and GTP/GDP ratios (see Pogolotti *et al*.^35^ and the section on Quality control in *Supplementary Methods*) and benchmarked the method against recovery-corrected whole broth sampling. We found that neither of these approaches overcome the effect of centrifugation, and the ATP/ADP ratios plummet from 10 down to 1 (Supplementary Figure 5a). We conclude that centrifugation is not a suitable sample collection method for nucleotide measurements – or at least requires further optimization.

The other commonly used harvesting technique is rapid filtration^22, 27, 36^ (Fig. 1). 10–40 ml cultures were filtered through a 0.45 µm membrane filter using a vacuum pump, and the filters were immediately transferred into Eppendorf tubes that contained ice-cold acid. We tested both formic and acetic acid, and found that the latter provides a stronger signal for all nucleotides, most likely due to having a better extraction efficiency and/or recovery (Supplementary Figure 5c). Eppendorf tubes, together with the filters, cell mass, and acid they contained, were snap-frozen in liquid nitrogen and stored at −80 °C. Extraction, with occasional vortexing, was performed for 30 minutes after the samples were thawed on ice and it relies on the acid already present in the samples. Finally, the filter was removed, and the samples freeze-dried. We second the advice given by Nazar and colleagues^36^ that one should not remove the cell pellet prior to freeze-drying because extraction completes during the freeze-drying step (Supplementary Figure 5d).

We quantified the recoveries by adding nucleotide standards (AMP, ADP and ATP; GMP, GDP, GTP, and ppGpp) to frozen samples and subjecting them to sample processing (Table 1). The recoveries are all above 80% with the exception of GTP (66%) and ppGpp (46%). The following sections all employed harvesting by filtration.

### Nucleotide pools in *E. coli* throughout the growth curve and during acute stringent response

The final version of our method utilizes harvesting by rapid filtration, followed by acid extraction and freeze-drying. The nucleotide species were chromatographically resolved and quantified using a combination of C18 IPRP-HPLC (GMP unresolved and co-migrating with IMP; GDP and GTP; AMP, ADP and ATP; CTP; UTP) and SAX in isocratic mode (ppGpp and pppGpp). To benchmark the method we quantified the nucleotide pools in *E. coli* BW25113^37^ both throughout the growth curve and upon acute stringent response. Bacterial cultures were grown in MOPS medium supplemented with 0.4% glucose^38^ at 37 °C with vigorous shaking.

**Figure 4:**
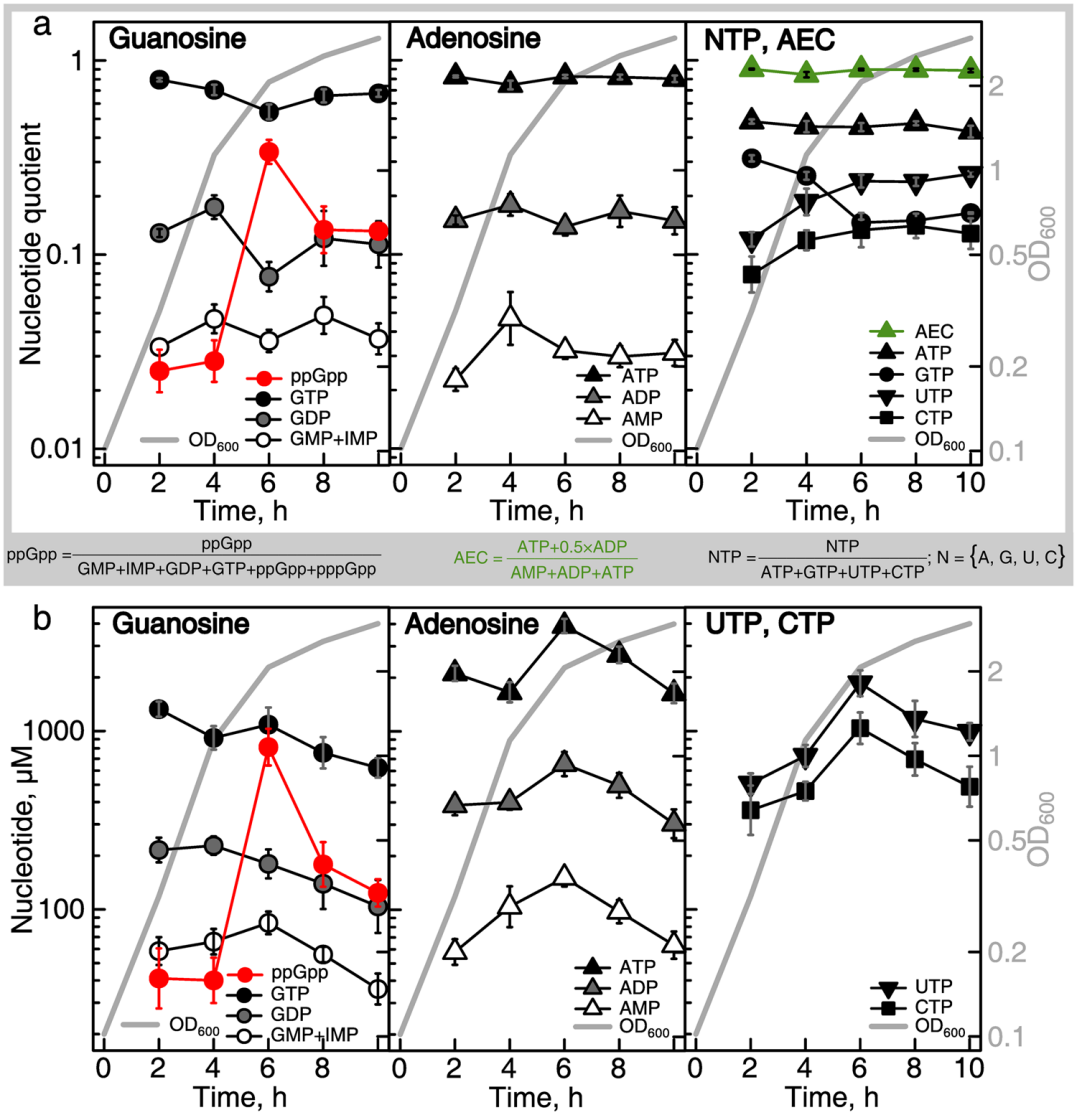
With the exception of ppGpp, the levels of nucleotides are stable in *E. coli* throughout the growth curve. (**a**) Intracellular nucleotides of *E. coli* cultures grown in defined minimal medium (MOPS 0.4% glc at 37 °C with vigorous aeration) are expressed as ratios of guanosine, adenosine, and NTP pools as indicated in the insert. AEC refers to the adenylate energy charge defined by Atkinson^7^. Cells were harvested by filtration and nucleotides extracted with acetic acid. ppGpp was measured using isocratic SAX and the remaining nucleotide species were quantified using gradient IPRP. GMP was not resolved using IMP and the earliest feasible sampling point was at an OD_600_ of 0.3. (**b**) Intracellular nucleotide levels are expressed as absolute concentrations. The cell volumes used to convert from nucleotide ratios to absolute concentrations were estimated using a method provided in *Supplementary Methods*. Error bars indicate the standard error of the mean of biological replicates (n = 7).

First, we quantified how the nucleotide levels change across the growth curve. Our experimental pipeline, even after optimization, still requires a considerable amount of starting material. Approximately 5.0 OD_600_ units for unstressed cells per measurement are required to detect ppGpp. Housekeeping nucleotides can be measured using as little as 1.0 OD_600_, however, in this report we were specifically interested in quantifying ppGpp as well. Therefore, the earliest time point was at OD_600_ 0.3 during the mid-logarithmic growth phase, followed by sampling every second hour for 8 hours until the stationary phase at OD_600_ 3.0. At that point, growth slows down and yields less than a 10% increase in OD_600_ per hour. The samples were processed as described above and the nucleotide levels were expressed as quotients (Fig. 4a) or as absolute concentrations of individual nucleotide species (Fig. 4b and Table 2). The quotients were calculated either as relative fractions of corresponding nucleotide species in the guanosine or adenosine pools (Fig. 4a left and middle panels, respectively) or as fractions of different triphosphate species in the triphosphate pool (Fig. 4a right panel). For exact numerical values of the data see Table 2.

**Table 2:** Nucleotide pools in *E. coli* throughout the growth curve. *E. coli* cultures were grown in defined minimal MOPS medium supplemented with 0.4% glucose at 37 °C with vigorous aeration and samples were removed for nucleotide measurements. The cell volumes necessary for calculation of absolute concentrations were estimated using a method provided in *Supplementary Methods*. The data are plotted as a function of time on (Fig. 4). Growth phase specific values are provided for guanosines and pyrimidines because the quotient was less stable than that for adenosines. When growth phase is not specified, the number provided corresponds to the mean value over all growth phases. 95% CI: 95% confidence interval. *NA*: not available.

Nucleotide		Quotient	Concentration
		mean_[95% CI]_, %	mean_[95% CI]_, µM
Adenosines	ATP	80_[77; 83]_	2200_[1900; 2600]_
	ADP	16_[14; 18]_	430_[370; 500]_
	AMP	3.2_[2.7; 3.8]_	88_[72; 110]_
Guanosines	GTP	67_[62; 72]_	900_[760; 1100]_
	* exponential*	75_[69; 81]_	1100_[870; 1400]_
	* transition*	54_[44; 67]_	1100_[630; 1900]_
	* stationary*	67_[61; 73]_	690_[530; 880]_
	GDP	12_[9.7; 15]_	160_[130; 200]_
	* exponential*	15_[13; 18]_	220_[180; 270]_
	* transition*	7.7_[4.9; 12]_	180_[110; 290]_
	* stationary*	12_[7.5; 18]_	120_[73; 200]_
	GMP + IMP	4_[3.4; 4.7]_	58_[49; 69]_
	* exponential*	3.9_[3.1; 5.0]_	62_[48; 80]_
	* transition*	3.6_[2.6; 5.0]_	84_[58; 120]_
	* stationary*	4.3_[3.1; 5.9]_	46_[35; 59]_
	ppGpp	8.1_[5.2; 13]_	120_[72; 200]_
	* exponential*	2.7_[1.8; 3.9]_	40_[25; 67]_
	* transition*	34_[23; 50]_	810_[420; 1600]_
	* stationary*	13_[9.7; 18]_	150_[100; 220]_
NTPs	CTP	12_[10; 14]_	570_[450; 720]_
	* exponential*	7.9_[4.7; 13]_	360_[160;780]_
	* stationary*	13_[11; 16]_	710_[530; 950]_
	UTP	20_[18; 23]_	970_[790; 1200]_
	* exponential*	12_[9.6; 15]_	510_[360; 710]_
	* stationary*	24_[23; 26]_	1300_[1000; 1600]_
	ATP	46_[44; 48]_	
	GTP	20_[18; 23]_	
	* exponential*	28_[26; 31]_	
	* transition*	15_[13; 16]_	
	*stationary*	16_[15; 16]_	
AEC	ATP, ADP, AMP	88_[86; 90]_	

The guanosine pool is dominated by GTP which constitutes 60–80% of the total pool (dropping to 54% when ppGpp is at its peak). GDP levels fluctuate at around 12%, and GMP combined with IMP constitute around 4% of the pool. The level of ppGpp undergoes a dramatic change: upon the entry into stationary phase (OD_600_ = 2.0) its fraction in the guanosine pool increases from 3% to 34% and remains elevated at 13% in the stationary phase. The adenosine pools remain stable across the growth curve, with ATP constituting 80%, ADP 16% and AMP 3–4%. This results in the AEC staying around 0.88. Quantification of the nucleotide triphosphate pool quotients across the growth curve shows that while ATP is stable at 46%, the GTP quotient drops from 28% to 16% of the total NTP species. The drop is reciprocated by an increase in the UTP and CTP quotients from 12% to 24% and from 8% to 13%, respectively.

We also attempted to estimate the absolute concentrations of intracellular nucleotides (Fig. 4b and Table 2). To estimate the cell concentration we employed flow cytometry. To estimate the cell volume, we used light microscopy and approximated the cell shape as a cylinder with two hemispherical caps (see *Supplementary Methods* for details). The cell volume decreases from 1.2 ± 0.3 in the exponential phase to 0.5 ± 0.2 femtoliters in the stationary phase (Supplementary Table 1 and Supplementary Figure 7). However, such estimates are prone to systematic errors because they rely on unverified *ad hoc* assumptions, such as that the periplasm to cytoplasm volume ratio remains unchanged across the growth curve. Moreover, fixation and staining could introduce experimental artifacts. The concentration of the nucleotides change across the growth curve in a similar manner to the nucleotide fractions. The ppGpp levels are around 40 μM during the exponential phase, increase up to 800 μM upon the entry to stationary phase, and then stabilize at 150 μM. GTP levels steadily decline from 1100 μM in the mid-logarithmic growth phase to 700 μM in the stationary phase. The ATP levels fluctuate from 1600 to 3900 μM. CTP and UTP vary from 360 to 710 μM and 510 to 1300 μM, respectively.

**Figure 5:**
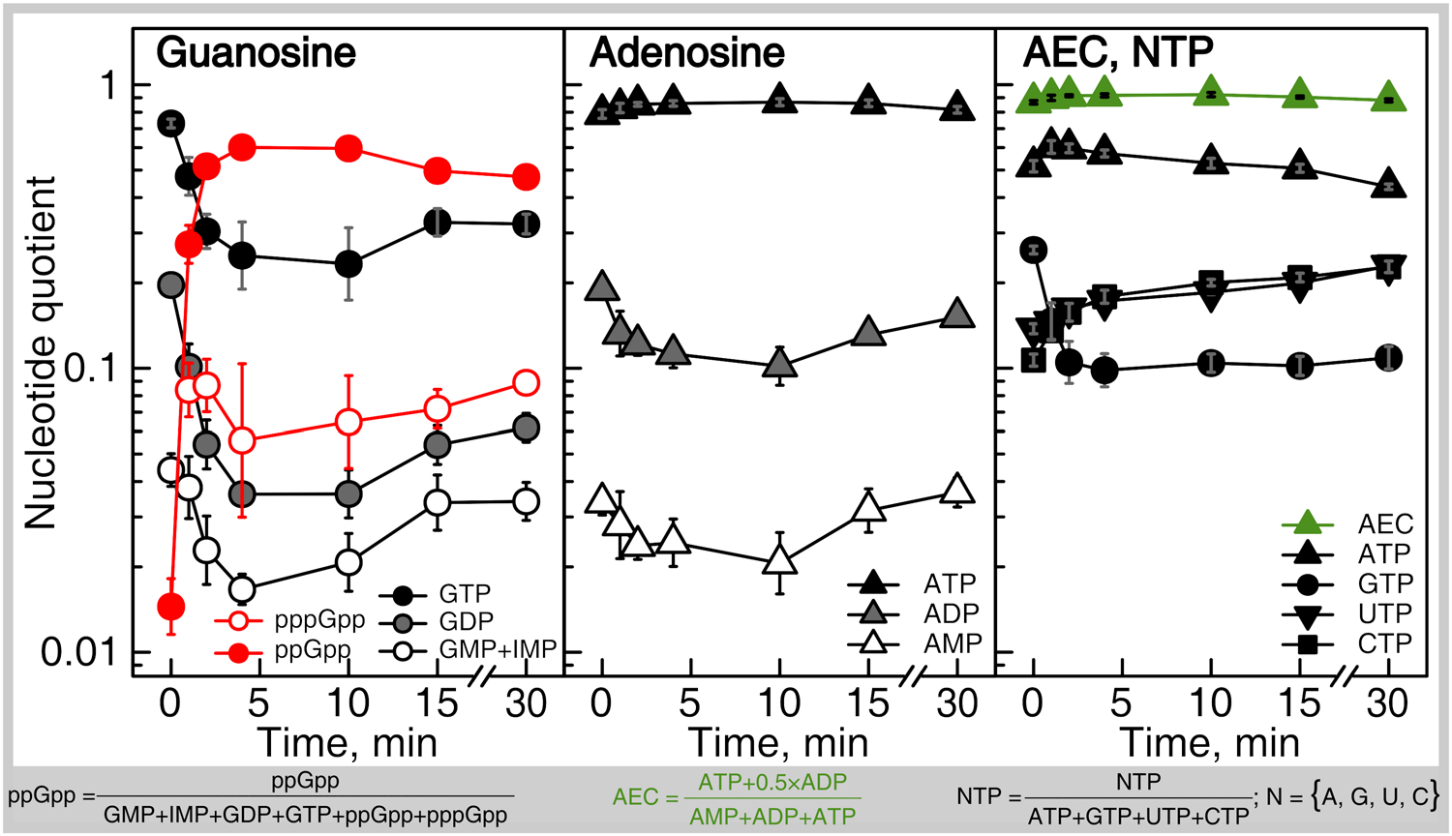
Kinetics of nucleotide upon mupirocin-induced stringent response. Intracellular nucleotides of *E. coli* cultures growing in defined minimal medium (MOPS 0.4% glc at 37 C with vigorous aeration) were measured both prior to and after the addition of mupirocin (3-times the MIC, 150 µg/ml, added when cells reached OD_600_ 0.5) at time point zero. The nucleotide concentrations are expressed as ratios of the guanosine, adenosine, and NTP pools as indicated in the insert; AEC stands for adenylate energy charge. Cells were harvested by filtration and the nucleotides were extracted with acetic acid. ppGpp and pppGpp were measured using isocratic SAX and the remaining nucleotides were measured using gradient IPRP. pppGpp was below the limit of detection in unstressed cells (zero time point). Error bars indicate the standard error of the mean of biological replicates (n = 8 at timepoint zero, otherwise n = 3–4). For numeric representation of the data see Table 2.

We went on to quantify the nucleotide dynamics upon the induction of an acute stringent response. A bacterial culture was grown to OD_600_ 0.5 and the stringent response was induced by addition of a completive inhibitor of isoleucine aminoacyl-tRNA synthetase – the antibiotic mupirocin (pseudomonic acid)^25^ – to a final concentration of 150 μg/ml (3 × MIC). Samples were taken over a time course (1, 2, 4, 10, 15 and 30 minutes), analyzed, and the nucleotide quotient was plotted as a function of time (Fig. 5). For exact numerical values of the data see Table 3.

**Table 3:** Nucleotide pools in *E. coli* during stringent response. *E. coli* cultures were grown in defined minimal medium MOPS supplemented with 0.4% glucose at 37 °C with vigorous aeration until OD_600_ 0.5. Then, stringent response was induced by mupirocin at 150 µg/ml (3 × MIC), and samples were withdrawn for nucleotide measurements at times indicated. The data are plotted as a function of time on (Fig. 5). 95% CI: 95% confidence interval. *NA*: not available.

Nucleotide		Quotient: mean_[95% CI]_, %						
		0 min	1 min	2 min	4 min	10 min	15 min	30 min
Adenosines	ATP	79_[73;86]_	83_[73;93]_	85_[81;90]_	86_[80;92]_	87_[80;94]_	86_[80;93]_	82_[77;87]_
	ADP	19_[16;23]_	13_[7.4;24]_	12_[9.1;16]_	11_[7.8;16]_	10_[6.6;16]_	13_[11;15]_	15 _[13;18]_
	AMP	3.4_[2.7;4.3]_	2.8_[1.2;6.7]_	2.4_[1.7;3.4]_	2.4_[1.3;4.5]_	2.1_[1.0;4.1]_	3.2_[1.9;5.2]_	3.7_[2.8;4.8]_
Guanosines	GTP	73 _[67;79]_	48_[25;92]_	30_[17;55]_	25_[7.7;81]_	23_[9.2;59]_	33_[23;47]_	32_[27;39]_
	GDP	20_[16;25]_	10_[4.6;22]_	5.4_[2.3;13]_	3.6_[2.7;4.8]_	3.6_[1.9;6.7]_	5.4_[3.3;8.9]_	6.2_[4.8;8.0]_
	GMP + IMP	4.4_[3.2;6.0]_	3.8_[1.3;11]_	2.3_[0.69;7.6]_	1.7_[0.98;2.8]_	2.1_[0.98;4.4]_	3.4_[1.7;6.9]_	3.4_[2.4;4.9]_
	ppGpp	1.4_[0.85;2.5]_	27_[14;53]_	52_[37;72]_	60_[49;74]_	60_[45;78]_	50_[40;61]_	47_[43;52]_
	pppGpp	*NA*	8.4_[3.3; 21]_	8.7_[3.5; 22]_	5.6_[0.38;81]_	6.5_[2.0;21]_	7.2_[4.4;12]_	8.9_[7.5;10]_
NTPs	CTP	11_[9.5;12]_	14_[11;17]_	16_[13;20]_	18_[15;21]_	20_[19;22]_	21_[19;23_]	23_[21;25]_
	UTP	14_[13;15]_	15_[13;17]_	16_[14;19]_	17_[16;19]_	19_[18;19]_	20_[18;22]_	23_[22;24]_
	ATP	52_[46;57]_	60_[51;71]_	60_[53;67]_	57_[52;63]_	53_[47;59]_	51_[46;56]_	44_[42;46]_
	GTP	26_[24;28]_	15_[7.6;28]_	10_[5.0;22]_	9.8_[5.5;18]_	10_[8.2;13]_	10_[8.0;13]_	11_[8.8;13]_
AEC	ATP, ADP, AMP	87_[84;90]_	90_[84;96]_	91_[89;94]_	92_[88;96]_	92_[88;97]_	90_[88;93]_	88_[86;91]_

The dynamic changes are rapid and stabilize within the first 4 minutes. The level of (p)ppGpp increases dramatically, raising from 1.4% to 60%. ppGpp becomes the most abundant guanosine nucleotide, surpassing GTP which drops from 73% to 25% reciprocating the kinetics of (p)ppGpp accumulation. pppGpp, which is undetectable in untreated cultures, increases to about 8% and becomes more abundant than GDP which drops from 20% to 4%. The adenosine pools remain stable and the AEC remains near 0.9. The balance of the NTP pool undergoes a change similar to that upon transition from the exponential to stationary phase. ATP remains stable at 54%. The UTP and CTP fractions increase from 14% to 23% and 11% to 23%, respectively. The GTP quotient rapidly drops from 26% to 10%.

## Discussion

Here we describe an HPLC-UV method capable of analyzing the nucleotide content of about 50 biological samples in three days. The required sampling volume is typically 10 ml, but must be increased to 30–50 ml when analyzing (p)ppGpp levels in rapidly growing cells (doubling time ≤1 h). Rapid vacuum filtration is an optimal method for cell harvesting. Sampling by centrifugation dramatically perturbs the triphosphate pools and is thus not applicable. Our extraction procedure is nearly identical to Nazar *et al*.^36^, and, like them, we favor extraction with acetic acid over the more commonly used formic acid. Our IPRP-HPLC gradient method is nearly identical to the one implemented by Payne and Ames^22^ and provides excellent sensitivity and resolution when quantifying most ‘housekeeping’ nucleotides: GDP and GTP; AMP, ADP and ATP; CTP; UTP; GMP is unresolved and co-migrates with IMP. Since IPRP-HPLC is unsuitable for measuring (p)ppGpp (reported here and elsewhere^39^), we complement it with SAX-HPLC in isocratic mode.

We employed our method to study an *E. coli* BW25113 K12-strain both throughout the growth curve and during acute stringent response induced by mupirocin (pseudomonic acid), a competitive inhibitor of isoleucine aminoacyl-tRNA synthetase. Our results from steady state exponential phase cells align well with a multitude of earlier reports^14, 20, 40–48^ (Supplementary Figure 8), and provide, to our knowledge, the most comprehensive description of how the bacterial nucleotide pools change across the growth curve. We employed the very same method to study both bacteria transitioning from exponential to stationary phase and undergoing acute starvation. The nucleotides were quantified as quotients of pools that compete for the same key biological targets: i) the guanosine pool, ii) the adenosine pool and iii) the nucleotide triphosphate (NTP) pool (Figs. 4 and 5; Tables 2 and 3). The ratio of guanosine species affects numerous GTPase enzymes for which the GTP acts as a substrate, and other species, notably (p)ppGpp, act as orthosteric inhibitors^49–51^. Translation is inhibited by (p)ppGpp outcompeting GTP while binding to translational GTPases, such as initiation factor IF2 and elongation factor EF-G^50, 52^. The ribosome assembly is inhibited in a similar manner by targeting the assembly factors EngA, RsgA, RbgA, Era, HflX and ObgE^51, 53, 54^. In addition, (p)ppGpp specifically targets numerous enzymes, notably *E. coli* RNAP^2, 55^. The adenylate pool regulates enzymes that are sensitive to changes in the adenylate energy change, AEC^56^. Recently, the ATP/ADP ratio was suggested to throttle protein synthesis via ribosome-associated ABCF ATPase EttA/YjjK^57^. The NTP pool regulates transcription of ribosomal RNA (rRNA) by altering the ratio of GTP and ATP initiator nucleotides^58, 59^.

The nucleotide quotients are stable across growth curve, with two exceptions: i) the spike in the ppGpp fraction within the guanosine pool upon the entry to stationary phase, and ii) concomitant decrease of the GTP fraction in the NTP pool (Fig. 4a; Table 2). The former effect is likely to be the cause of the latter: ppGpp is known to throttle the production of guanosine nucleotides in *E. coli* via direct inhibition of IMP dehydrogenase^60^. While the GTP fraction in the NTP pool decreases, the ratios of the housekeeping guanosine nucleotides are stable, i.e. GTP/GDP/IMP + GMP remain as 7 ± 2/1 ± 0.1/0.4 ± 0.06 (Fig. 4a; Supplementary Figure 9a). The adenosine pool also remains stable at an ATP/ADP/AMP ratio of 6 ± 0.7/1 ± 0.06/0.2 ± 0.03. When we induce the acute stringent response by mupirocin, the (p)ppGpp levels increase dramatically and reach near-maximum levels already in the first time point at one minute (Fig. 5). This increase is reciprocated by a drop in the levels of the other guanosine species. After four minutes of treatment, the system reaches a steady state with the guanosine pool dominated by ppGpp, which becomes not only more abundant than GTP, but more abundant then all of the housekeeping guanosine nucleotides combined. At the same time, the relative ratio of GTP/GDP/IMP + GMP, although somewhat less steady than during growth curve (Supplementary Figure 9b), generally remains at 6 ± 0.8/1 ± 0.3/0.5 ± 0.08, similarly to the ratios across the growth curve. As with the adenosine ratio, ATP/ADP/AMP remains stable at 6 ± 0.5/1 ± 0.03/0.2 ± 0.02.

Our analysis highlights two overarching principles. First, both the guanosine and adenosine housekeeping nucleotides maintain the NTP/NDP/NMP ratio at 6 ± 0.5/1 ± 0.03/0.3 ± 0.03 both during a typical batch culture growth trajectory and upon acute amino acid starvation. The concentration of these NTPs are instrumental for operation of the cell and are, most likely, maintained at these levels in order to ensure viability. Second, while the ppGpp and pppGpp levels vary drastically (40- and ≥8-fold, respectively), these changes are decoupled from the stable quotients of the housekeeping pool. This separation of housekeeping and signaling functions might break down at a shorter time scale, i.e. below one minute. However, following the relaxation kinetics upon perturbation is not possible with our method because the temporal resolution is not sufficient. Following these rapid changes would require one to develop a dedicated experimental approach.

## Methods

For a detailed description of the experimental procedures, see *Supplementary Methods*. We grew *E. coli* strain BW25113^37^ in MOPS medium supplemented with 0.4% glucose^38^ incubated at 37 °C while shaking at 200 rpm. Overnight cultures (1–2 ml) in glass test tubes shaken at an angle were used to initiate each experiment. To obtain nucleotide measurements over the growth curve, overnight cultures were diluted to OD_600_ 0.1 (about 30-fold dilution) and grown in 80 ml of pre-warmed medium in 500 ml conical flasks. Samples for nucleotide quantification were taken at 2, 4, 6, 8, and 10 hours. To estimate the cell size, cells were stained with 1% nigrosin and spread onto a microscope slide, air dried and imaged using oil-immersion light microscopy. Pixel-to-µm conversion was performed using microspheres 1 µm in diameter for calibration and image analysis was performed using ImageJ^61^. 100–150 cells were measured for each time point across the growth curve. Bacterial cell concentration was determined by fixing the cell culture (60 µl of cell culture with 20 µl 10% paraformaldehyde) and storing samples at −80 °C until further analysis using a flow cytometer (LSRII, BD Biosciences). To study the stringent response, overnight cultures were diluted 100-fold in 100 ml of pre-warmed medium in a 1 l conical flask and grown to OD_600_ 0.5. Then, an initial sample was taken and the stringent response was evoked at time zero by adding mupirocin at 150 µg/ml (3 × MIC). Samples were then taken at 1, 2, 4, 10, 15 and 30 minutes. In both experiments, the volume of culture used to quantify the nucleotides was 3 × 10 ml for first timepoint and 10 ml for the remaining timepoints. All sampling and sample processing was performed using the filtration technique that is thoroughly described in *Supplementary Methods*. All nucleotides were quantified using IPRP-HPLC, except ppGpp and pppGpp which where quantified using isocratic SAX-HPLC. Reference nucleotide standards were from Thermo Fisher Scientific and Sigma-Aldrich except ppGpp and pppGpp which were in-house synthesized as described elsewhere^62^.

The datasets generated during the current study that were not included in tables, are available from the corresponding authors on reasonable request.

## Electronic supplementary material

Supplementary Information
