# Supplementary material for: HPLC-based quantification of bacterial housekeeping nucleotides and alarmone messengers ppGpp and pppGpp
Source: Sci Rep. 2017 Sep 8;7:11022. doi: 10.1038/s41598-017-10988-6 (PMC5591245; doi:10.1038/s41598-017-10988-6)
Supplement: Supplementary file 1 — Supplementary Information [file 41598_2017_10988_MOESM1_ESM.pdf]

Supplementary Information

HPLC-based quantification of bacterial housekeeping nucleotides and alarmone messengers ppGpp and pppGpp

Vallo Varik[§,1,2,3,⌘], Sofia Raquel Alves Oliveira[1,⌘], Vasili Hauryliuk[§,1,2,3] and Tanel Tenson[§,1]

[1]University of Tartu, Institute of Technology, Nooruse 1, 50411 Tartu, Estonia

[2]Department of Molecular Biology, Umeå University, Building 6K, 6L University Hospital Area, SE-901 87 Umeå, Sweden

[3]Laboratory for Molecular Infection Medicine Sweden (MIMS), Umeå University, Building 6K and 6L, University Hospital Area, SE-901 87 Umeå, Sweden

[§]denotes the corresponding authors

[⌘]denotes equal contribution

Contact details of corresponding authors:

Vallo Varik: vvarik.mail@gmail.com, +372 526 4034

Vasili Hauryliuk: Vasili.hauryliuk@umu.se, +46 90 785 08 07

Tanel Tenson: tanel.tenson@ut.ee, +372 737 4844

CONTENTS

Supplementary Text 3
Introduction 3
Sample acquisition 3
Nucleotide extraction 5
Nucleotide quantification 8
Quality control 9
Discussion 10
Supplementary Methods 13
Nucleotide Samples for HPLC—A Whole Culture Approach 13
Nucleotide Samples for HPLC—Harvest by Filtration Approach 15
Strong Anion Exchange HPLC 18
Ion-Paired Reverse Phase HPLC 22
Bacterial Cell Number and Volume Estimations 25
Data Handling and Analysis 25
Supplementary Figures 27
Supplementary Figure 1 27
Supplementary Figure 2 28
Supplementary Figure 4 31
Supplementary Figure 5 32
Supplementary Figure 6 34
Supplementary Figure 7 35
Supplementary Figure 8 37
Supplementary Figure 9 39
Supplementary Figure 10 41
Supplementary Tables 42
Supplementary Table 1 42
Supplementary References 43

SUPPLEMENTARY TEXT

Introduction

We report here a workflow we have applied successfully, besides *Escherichia coli* to cultures of *Bacillus subtilis*, *Acinetobacter baumannii*, *Pectobacterium wasabiae* and *Pseudomonas putida* (**Supplementary Figure 1d and Supplementary Figure 10a-d**). We took into account the comprehensive knowledge from the literature and chose HPLC-UV analysis due to its relative simplicity, availability, ability to detect rich set of nucleotides and applicability to analysis of non-growing bacterial cultures or the ones grown in complex growth media.

The workflow from bacterial culture to quantitative estimates of nucleotide concentration in the cell can be divided into several steps (**Figure 1**): (i) sample acquisition, (ii) extraction, and (iii) quantification. Below we provide an overview of existing techniques and associated pitfalls.

Sample acquisition
It is essential that cellular metabolism must be rapidly quenched during the preparation of a nucleotide extract, thus 'freezing' the biologically relevant status quo. This can be achieved either in the course of sample acquisition and extraction or, alternatively, during a dedicated quenching step. Sample acquisition is performed either by separating cells from culture medium or by sampling whole culture broth. Separation of cells from culture medium, i.e. harvesting, can be performed either by filtration or centrifugation and results in a stronger signal during the detection step due to the reduced sample size leading to an increased concentration of metabolites. However, addition of a relatively slow harvesting step poses a challenge for the detection of high flux metabolites, such as nucleotides in a rapidly growing *Escherichia coli* culture (Cole, Wimpenny et al. 1967, Chapman, Fall et al. 1971, Lundin and Thore 1975, Payne and Ames 1982, Bolten, Kiefer et al. 2007, Ishii, Nakahigashi et al. 2007, Buckstein, He et al. 2008, Bennett, Kimball et al. 2009). To overcome this problem, the Rabinowitz lab has developed an approach utilizing cultivation of bacteria on a nitrocellulose filters on top of the agarose plates, so that the filters can be rapidly transferred to extraction solution (Brauer, Yuan et al. 2006).

Centrifugation is not applicable for bacterial nucleotide measurements since it dramatically alters the nucleotide levels, i.e. highly phosphorylated compounds are converted to less phosphorylated species (Cole, Wimpenny et al. 1967, Chapman, Fall et al. 1971, Lowry, Carter et al. 1971, Lundin and Thore 1975, Payne and Ames 1982, Buckstein, He et al. 2008). Rapid vacuum filtration, on the other hand, gives reliable results for *B. subtilis* (Ochi, Kandala et al. 1981) and *E. coli* (Franzen and Binkley 1961, Nazar, Lawford et al. 1970, Bagnara and Finch 1972, Walker-Simmons and Atkinson 1977, Payne and Ames 1982). Washing, if necessary, is a critical step during filtration since a difference in ionic strength of a washing solution and of the growth medium can dramatically perturb the results (Bolten, Kiefer et al. 2007). Addition of a dedicated quenching step prior to harvesting is commonly used to counter the abovementioned challenges. The most frequent quenchers are cold 60% methanol in an aqueous buffer (Buchholz, Takors et al. 2001, Buchholz, Hurlebaus et al. 2002, Bolten, Kiefer et al. 2007, Hiller, Franco-Lara et al. 2007), cold aqueous solution of 0.9% NaCl (Wittmann, Kromer et al. 2004), formaldehyde (Little and Bremer 1982) and cold glycerol (Wittmann, Kromer et al. 2004). However, some of these approaches can perturb the consequent nucleotide measurements: leakage of the cytoplasm from bacterial cells was observed while using methanol (Wittmann, Kromer et al. 2004, Bolten, Kiefer et al. 2007) or 0.9% NaCl (although it was suggested to be due to cold shock, the effect of centrifugation can not be ruled out) (Wittmann, Kromer et al. 2004); a combination of formaldehyde quenching with alkali extraction tends to give a weaker signal and introduces a lot of variation (for details and references, see next section on nucleotide extraction).

All of the above-mentioned problems with harvesting can be avoided altogether by opting for a whole-culture broth sampling. In this case, quenching can be done by snap-freezing the sample with liquid nitrogen (Dominguez, Rollin et al. 1998, Chassagnole, Noisommit-Rizzi et al. 2002) or rapidly boiling it (Schaub, Schiesling et al. 2006). A more usual approach is transferring the cell suspension into an extraction solute. There are, however, several disadvantages of the whole culture broth approach. First, it results in considerably more dilute solution of metabolites. This necessitates either a highly sensitive detection method, such as counting radioactivity after labeling with ^{32}P via phosphate or nucleotide precursors, or additional enrichment steps, such as separation, precipitation, evaporation and/or freeze-drying. Second, nucleotides in extracellular and

intracellular material cannot be discriminated; performing an supplementary analysis of filtrate (Taymaz-Nikerel, de Mey et al. 2009) is a possible solution to this problem. An extreme example of this issue is quantification of nucleotide messenger cAMP which predominantly resides in the growth medium (Matin and Matin 1982). While 'housekeeping' nucleotides are not generally detected outside the cell (Lundin and Thore 1975, Taymaz-Nikerel, de Mey et al. 2009), it has been reported that *E. coli* cultures might accumulate AMP together with enzymes that affect nucleotide stability in the growth medium during stationary phase (Chapman, Fall et al. 1971). Third, components of growth media can interfere with the nucleotide measurements: inorganic salts can interfere with nucleotide detection by liquid chromatography (LC) coupled to UV detector (Bhattacharya, Fuhrman et al. 1995) or to mass-spectrometer (Bolten, Kiefer et al. 2007); organic components can inhibit enzymatic assays (Lundin and Thore 1975).

Nucleotide extraction

Extraction of nucleotides can be either mechanical (Meyer, Liebeke et al. 2010), using sonication (Lundquist and Olivera 1971), or by means of chemical treatment with hot (80-100 °C) or cold (≤4 °C) solutes. Hot extraction can be performed with ethanol (Lundin and Thore 1975, Taymaz-Nikerel, de Mey et al. 2009, Meyer, Liebeke et al. 2010), alkali (Lundin and Thore 1975, Cserjan-Puschmann, Kramer et al. 1999, Schneider, Murray et al. 2003), 23% chloroform (Dhople and Hanks 1973, Lundin and Thore 1975), water (Bagnara and Finch 1972, Bhattacharya, Fuhrman et al. 1995, Wittmann, Kromer et al. 2004, Hiller, Franco-Lara et al. 2007, Meyer, Liebeke et al. 2010) or aqueous buffer solutions (Lundin and Thore 1975, Hiller, Franco-Lara et al. 2007) (for a comprehensive comparison of extraction solutes see (Bagnara and Finch 1972, Lundin and Thore 1975, Meyer, Liebeke et al. 2010)). Ethanol is a reliable option for whole culture experiments (Lundin and Thore 1975, Taymaz-Nikerel, de Mey et al. 2009). Alkali lysis in a combination with quenching by aldehyde results, though not always (Lundin and Thore 1975), in relatively weak signal of nucleotides (Lundin and Thore 1975, Cserjan-Puschmann, Kramer et al. 1999, Schneider, Gaal et al. 2002, Schneider, Murray et al. 2003) and substantial variance (Schneider and Gourse 2004). Several artefacts can be speculated to be responsible. First, crosslinking of nucleotides to cellular material by aldehyde. It is plausible that aldehyde-alkali extraction might report the pool of free nucleotides i.e. soluble and not bound fraction. Second, degradation of nucleotides that

are instable in alkaline conditions, such as cyclic nucleotides (Markham and Smith 1952), (p)ppGpp (Cashel and Kalbacher 1970). Third, incomplete lysis which gives a systematic error, overrepresentation of the content of larger cells, and obtained results are therefore also a function of cell size distribution (Dennis, Ehrenberg et al. 2004).

At elevated temperatures, both enzymatic and chemical degradation of nucleotides are more likely. Cold extraction is therefore safer. It has its own drawbacks, however, a general and specific ones. Overarchingly, it does not necessarily completely stabilize the sample and nucleotides can still undergo a degradation even when kept at -10 to 4 °C (Holms, Hamilton et al. 1972, Lundin and Thore 1975, Rabinowitz 2007). This issue can be addressed by (1) chelation of Mg^{2+} and other divalent ions—common cofactors for enzymes—by addition of EDTA (Lundin and Thore 1975) (however, this is likely to interfere with detection on HPLC, see our Supplemental Materials and Methods); (2) acidifying the solution (Rabinowitz and Kimball 2007); (3) acid precipitating the enzymes and removing precipitate, preferably before any neutralization (Lundin and Thore 1975); and (4) keeping the samples sufficiently cold i.e. working on ice and storing at -20 °C. Cold extraction is performed most commonly with chloroform (Coulier, Bas et al. 2006), ethanol (Cserjan-Puschmann, Kramer et al. 1999, Meyer, Liebeke et al. 2010), methanol (Meyer, Liebeke et al. 2010), acetonitrile (Au, Su et al. 1989), acidic acetonitrile-methanol-water (Rabinowitz and Kimball 2007), sodium formate at pH 3.4 (Cashel and Gallant 1968), and various acids (Franzen and Binkley 1961, Bagnara and Finch 1968, Nazar, Lawford et al. 1970, Fischer, Zimmerman et al. 1982).

If organic extraction is a must, triphosphates are better extracted with acetonitrile-methanol-water than methanol-water mixtures (Rabinowitz and Kimball 2007). Most common is still the acidic extraction with perchloric (PCA) (Franzen and Binkley 1961), trichloroacetic (TCA) (Smith and Maaloe 1964), formic (Cashel and Gallant 1969, Bochner and Ames 1982) or acetic acid (Nazar, Lawford et al. 1970). The appeal of strong acids like TCA and PCA, compared to milder organic acids, lies in the fact that they are better at disruption of the cell envelope and quenching enzymatic activities: act fast and do not require freeze-thaw cycles to complete the extraction (Bagnara and Finch 1972). It is known, nevertheless, that some unidentified bacterial phosphatase activity can endure PCA (the activity can endure also boiling) and the best remedy is the addition of EDTA or removal of acid precipitate before neutralization (Lundin and Thore 1975). Yet strong

acids, especially if not kept sufficiently cold, are more prone to break down highly phosphorylated species (Cashel and Gallant 1968, Nazar, Lawford et al. 1970, Au, Su et al. 1989) and accordingly, (p)ppGpp is not efficiently extracted from biological material with PCA and TCA (Cashel 1969). Cold formic acid extraction is therefore by far the most common solvent for (p)ppGpp extraction, even though it also has been claimed to introduce artifacts via ppGpp degradation to ppGp (Lagosky and Chang 1978) (This is part of the motivation why less used lysozyme (Lagosky and Chang 1978), and alkali extraction (for that, see discussion above) (Little and Bremer 1982) were devised for ppGpp quantification). Regardless of the acid of choice, prolonged incubation in acidic conditions during extraction or analysis (pH 4-6 will suffice if working at room temperature), results in degradation of NADPH and NADH (Lowry, Passonneau et al. 1961) and various resultant degradation products might interfere with detection and/or quantification (reduced forms, on the other hand, are stable in acid but not in alkaline conditions (Kaplan, Colowick et al. 1951)).

It is often desirable to get rid of the acid. Chemical lability is the major concern, phosphoanhydride bonds are stable in acidic conditions only if kept sufficiently cold. There are three options: neutralization, extraction or evaporation. Perchloric acid has often been the acid of choice because of the ease of removal via neutralization: addition of KOH or K_2CO_3 will lead to precipitation of poorly water soluble $KClO_4$. It has been estimated, however, that after neutralization and removal of insoluble $KClO_4$, about 60 mM of salt remains in the solution (Pogolotti and Santi 1982). Accordingly, both PCA and TCA have been reported to interfere with downstream detection on HPLC (Au, Su et al. 1989, Gebelein, Merdes et al. 1992, Buchholz, Takors et al. 2001). It is likely that this interference can be surmounted using extraction of acid instead of neutralization (Khym 1975, Arezzo 1987). Amine-freon extraction separates the acid which forms a salt with amines and is partitioning into the freon phase, leaving the nucleotides in the aqueous phase (Khym 1975). TCA, soluble in ethyl ether, can be removed by repeated extraction with ether (Arezzo 1987). Instead of acid removal, nucleotides themselves could be extracted with either acid washed charcoal (Norit A) (Fiske 1934, Cabib, Leloir et al. 1953) or with ion exchange (Buckstein, He et al. 2008). Finally, volatile acids—such as TCA, formic, and acetic acid—can be removed by freeze-drying.

If the acid treatment was adequately strong—and/or acid precipitate removed before neutralization (see above)—to eliminate all enzymatic activities, neutralized aqueous nucleotide sample is often stable enough to be dried in centrifugal evaporator at room temperature (Khym 1975).

Nucleotide quantification

The last step is quantification of extracted nucleotides (**Figure 1**). This step is customarily based on liquid chromatographic separation. The alternatives to chromatography include enzymatic assays for adenosine nucleotides (Chapman, Fall et al. 1971, Schneider and Gourse 2004) or dNTPs (Solter and Handschumacher 1969, Lindberg and Skoog 1970, Skoog 1970), and capillary electrophoresis (Soga, Ueno et al. 2002, Markuszewski, Britz-McKibbin et al. 2003). Enzymatic assay of dNTPs and capillary electrophoresis are not common for nucleotide detection. Luciferase assay of adenosine nucleotides, however, established the concept of adenylate energy charge (Atkinson 1968) and laid the foundations for all nucleotide quantifications that followed: energy charge is a crucial measure of sample acquisition, extraction and/or storage quality.

The most widely used chromatographic technique for nucleotide separation is thin layer chromatography (TLC) (Bochner and Ames 1982). TLC relies on isotope (^{32}P) labeling and is thus confined to actively growing cells and media where the concentration of phosphate can be readily manipulated to facilitate efficient uptake and labelling. Note that stringent response, for example, is known to interfere with the uptake of phosphate and thus incorporation of the radioactive label (Edlin and Neuhard 1967, Gallant and Cashel 1967, Cashel and Gallant 1968, Irr and Gallant 1969). High performance liquid chromatography (HPLC), however, is free of those limitations of labelling. HPLC can rely on photometric (Little and Bremer 1982, Payne and Ames 1982) or mass spectrometric detection (Buchholz, Takors et al. 2001, Soga, Ueno et al. 2002, Bennett, Yuan et al. 2008, Meyer, Liebeke et al. 2010). There are three well-established HPLC modes to separate nucleotide mixtures: ion exchange, reverse phase and ion-paired reverse phase. Ion exchange is the first LC approach developed for nucleotides (Cohn 1949), has gained a lot of popularity and works especially fine for highly charged nucleotides (tri-, tetra- and pentaphosphates). Reversed phase is suitable only for nucleosides and cyclic nucleotides (Payne and Ames 1982). To increase the retention of highly charged nucleotides on

reversed phase column, a zwitterion, customarily tetrabutylammonium, is added to the mobile phase and the result is called ion-paired reverse phase (Hoffman and Liao 1977, Little and Bremer 1982, Payne and Ames 1982, Mack, Reed et al. 1985).

Next, resolved nucleotides need to be quantified. UV absorbance is a feasible, less expensive and more often available, whereas MS excels in sensitivity and accurate identification. The highly charged nature of nucleotides, however, poses a challenge for analysis on LC-MS. First, high salt concentrations of strong anion exchange, necessary to elute charged nucleotides, are not easily compatible with electrospray ionization (ESI). Second, nucleotides are not easily resolved on reverse phase, the most frequent and compatible LC mode for MS. Third, tetrabutylammonium salts of IPRP are not volatile and thus, again, not compatible with ESI. The best option therefore available is to use IPRP with either tributylamine (Luo, Groenke et al. 2007) or primary amine (Coulier, Bas et al. 2006) as a zwitterion. Hydrophilic interaction chromatography (HILIC) can also be attempted, preferably with an aminopropyl column (Bajad, Lu et al. 2006), but gives inferior separation as compared to IPRP.

Quality control

Relative levels of adenylate nucleotides — ATP, ADP and AMP — serve as a quality control for sampling, extraction and sample storage. ATP has an exceedingly high turnover rate with a half-life of around one-tenth of a second (Holms, Hamilton et al. 1972, Walsh and Koshland 1984) and therefore its levels report the efficiency of quenching enzymatic activity. ATP, same as other di- and triphosphates, can be further degraded chemically since phosphoanhydride bond is not stable in acid unless kept cold. One can readily spot the ATP degradation by assessing the ‘adenylate energy charge’ (AEC). AEC is calculated as

$$AEC = \frac{ATP + \frac{1}{2}ADP}{ATP + ADP + AMP}$$

and it falls between 0.75-0.95 for rapidly growing cultures, be it bacterium *E. coli* (Chapman, Fall et al. 1971) or yeast *Saccharomyces cerevisiae* (Ball and Atkinson 1975). A technical problem with AEC is that it requires concentrations of all the three adenylate species—ATP, ADP and AMP—and determining the relatively low levels of AMP can be

challenging. Fortunately, a simpler parameter — ATP/ADP ratio — is nearly as informative as AEC; for values below 5 caution should be exercised and values above 10 are desirable as discussed by Pogolotti and colleagues (Pogolotti and Santi 1982). The biochemical and/or biological justification comes from the fact that the ATP/ADP ratio is a key physiological regulator of the glycolytic flux in *E. coli* (Koebmann, Westerhoff et al. 2002) and it was recently suggested to regulate protein synthesis via ribosome-associated ABCF ATPase EttA/YjjK (Boel, Smith et al. 2014).

Discussion

The broadest scope of bacterial nucleotides from steady state exponential growth conditions are reported by Bochner and Ames (Bochner and Ames 1982), Bennett *et al.* (Bennett, Kimball et al. 2009) and Buckstein *et al.* (Buckstein, He et al. 2008) (**Supplementary Figure 8**). Bochner and Ames have measured the nucleotide levels in *Salmonella typhimurium* (rather than *E. coli*, the model organism characterized in the current report); however, due to conserved nature of central metabolism and exceptional breadth of the nucleotide species quantified, the seminal report has become the textbook reference of bacterial nucleotide levels. The work by Bennett and colleagues is remarkable in that they quantify not only nucleotides but also numerous other metabolites (Bennett, Kimball et al. 2009). Finally, the study by Buckstein and colleagues is apparently closest to ours in that only they have followed the rather complete set of nucleotides from exponential phase into stationary (Buckstein, He et al. 2008).

For abundance of guanosine nucleotides, the results of Buckstein *et al*. and are similar to ours in that GTP levels are gradually getting lower when cells go from exponential to stationary phase, and ppGpp is highest during stationary (**Figure 4b**). Yet we observe bigger elevation in levels of ppGpp during growth stop, and have some evidence for decrease in the level of all guanosine species. Moreover, the maximum levels of ppGpp in their work seem to occur right after cells have stopped growing whereas in our case, they occur right before the growth stop. Some of the differences in guanosine species are most likely because we have accomplished a better sensitivity whereas in their work, both GDP and ppGpp signal, given the scale, appear small and close to zero. For adenosine nucleotides, there are even less similarities: we do see an increase in ADP during the

growth stop which is, however, very mild if it exists at all. In addition, we do not observe higher than stationary levels of ATP in exponential phase. Curiously, we get very similar overall shape of ATP and ADP levels except that, again, their results appear somewhat shifted along the growth curve towards later stationary. From that, in addition to the timing of ppGpp peak discussed above, it is also conceivable that we are over-interpreting some of the similarities by looking at the results which happened actually later in stationary phase in Buckstein and colleagues (Buckstein, He et al. 2008).

During the stringent response, accumulation of ppGpp precedes the curtailment of transcription (Cashel 1969, Gallant, Erlich et al. 1970). The initial drop in GTP must therefore result from allocation of guanosine pools in favor of (p)ppGpp. Next, when transcription—the major consumer of guanosines—stops, with all other things equal, GTP levels would be expected to expand whereas in fact they stay about half of the normal (**Figure 5** and references (Gallant and Harada 1969, Gallant, Erlich et al. 1970)). The fact that they do not expand suggests an inhibition of synthesis of GTP. Indeed, restriction of guanosine nucleotide biosynthesis by ppGpp is known from the literature (Gallant, Irr et al. 1971) and evident from our results (**Figure 4b**).

Besides drop in GTP and in ATP, the latter being often somewhat smaller, earlier reports of nucleotide levels during stringent response are contradictory. Edlin and Neuhard (Edlin and Neuhard 1967) report a gradual decline in all triphosphate pools to about a half within 30 min of stringent response. Both rapid shrinkage (Cashel and Gallant 1968) and increase (Edlin and Stent 1969) of UTP and CTP have been reported. Moreover, ATP and GTP stayed rather stable in according to Edlin and Stent (Edlin and Stent 1969). Also Edlin and Broda (Edlin and Broda 1968) record rather stable triphosphate pools except that GTP is clearly declining.

Most of the earlier work has been done with radioactive labeling—either with nucleosides or with $^{32}PO_4$. Possibly confounding the interpretation and/or explaining the contradictory results, in stringent strains at stress, phosphorylation of nucleotides is inhibited (Edlin and Neuhard 1967, Gallant and Cashel 1967, Cashel and Gallant 1968, Irr and Gallant 1969). In addition, stringent response can experimentally elicited in two different ways: (i) restraining the supply of amino acid or (ii) obstructing the

aminoacylation of tRNA. The former, in principle, can be somewhat alleviated by the turnover of protein whereas the latter cannot be mitigated.

SUPPLEMENTARY METHODS

Nucleotide Samples for HPLC—A Whole Culture Approach

Equipment and materials

(1) Manifold freeze-drier

Some simple manifold system will do, equipment does not have to be resistant to aggressive chemicals. If manifold freeze-dryer is not available, skipping the freeze-drying might be possible but we have not tried to do so.

(2) FPLC system with a peristaltic pump, column holder and UV detector

Place the system in 4 °C refrigerator or work in 4 °C room (it is crucial to keep your chromatography cold!)

(3) FPLC column with 1 mL QSepharose FF column matrix (GE Healthcare # 17051001)

(4) Waterbath

(5) Formic acid

(6) 0.2 µm syringe filters

(7) 2 M LiCl, 25 mM Tris pH 8 (about 100 mL)

Use 1 M Tris-HCl pH 8.0 and 8 M LiCl stock to prepare the buffer. No need to adjust or measure pH after mixing. To prepare the 8 M LiCl stock, weigh the salt, mix with water—it does not dissolve readily yet—bring to the final volume and mix some more until dissolved.

(8) 1 M KH_2PO_4 (about 50 µL)

(9) 96% EtOH

(10) 20% EtOH

Sample acquisition

(1) Harvest cells by pouring 10-40 mL of culture on ice-cold formic acid (1 M final) and quick freezing in liquid nitrogen

Work as quick as possible: (i) cell physiology is very fast to change (in response to slight shifts in temperature, oxygen etc.) and result in rapid alteration of metabolite levels, (ii) phosphoanhydride bonds are stable in formic acid only at low temperature. Use about 10-13 mL of E. coli culture of OD_{600} *0.5, however, for fast*

exponentially growing unstressed cells that much of an starting material gives small peak, so larger culture volumes might be necessary (30-50 mL)

(2) Store samples at -80 °C until extraction

Extraction

(1) Quick thaw in 37 °C water bath

Apply as much heat as needed to thaw, yet as little as possible to avoid chemical degradation. Shake or vortex samples couple of times during thaw to spread the heat evenly. If you want to assess your recovery of nucleotides, add known amount of nucleotide standard to the frozen sample before thaw.

(2) 30 min on ice with occasional vortexing

(3) Centrifuge at 5,000 G, 10 min, 4 °C

(4) Decant supernatant directly onto syringe with 0.2 µm filter, filtrate

Column concentration on Q Sepharose FF

(1) Equilibrate/wash column with water (we use ~6.5 mL/min)

(2) Dilute sample 20x in mQ (deionized water) water (usually for 10 mL sample, fill up to 200 mL with ice cold water in ice-cold bottle)

(3) Load 20x diluted sample onto Q-Sepharose FF column (~6.5 mL/min)

(4) Wash with cold mQ (2-3 min, ~6.5 mL/min)

(5) Elute 1 mL/min with 2 M LiCl, 25 mM Tris pH 8

Follow the elution at 254 nm, when the peak appears and start to collect (do not forget to account for dead volume, i.e. delay between reaching the detector as opposed to collector), then start to collect into 50 mL tube. In case of 10 mL initial sample, we collect a fraction of 4-5 mL

(6) Subject the fraction to precipitation procedure (see below, next section)

(7) Wash the column with 2 M LiCl, 25 mM Tris pH 8

(8) Equilibrate column with mQ water for next round

When finished, wash the column with water and store in 20% EtOH

Precipitation

(1) Add 4 volumes of cold 96% EtOH (ice-cold or -20 °C), 4 µL 1 M KH_2PO_4 (co-precipitate), followed by vortexing for 2-3 sec

(2) Transfer to -20°C for O/N precipitation or precipitate 10 min on ice

We mostly do an O/N precipitation, haven't really determined recoveries of shorter precipitation, however, shorter precipitation is expected to give good recoveries—about 80-90% (personal communication with Mike Cashel)

(3) Centrifuge at 5,525 G, 20 min, 4 °C using swing-out rotor, decant super

(4) Rinse pellet with 70% EtOH

The objective is to rinse the tube and pellet with a gentle swirl, do not (!) attempt to resuspend the pellet.

(5) Centrifuge at 5,525 G, 20 min, 4 °C using swing-out rotor, decant super

(6) Dry the pellet by freeze-drying (short freeze-drying will suffice, about 20 min)

Skipping the freeze-drying might be possible but we have not tried to do so.

(7) Resuspend the dried pellet in cold water (200 µL)

(8) Transfer to cold 1.5 mL microcentrifuge tubes

(9) Centrifuge for at least 30 min at max RPM, 4 °C

(10) Collect supernatant into new fresh tube

(11) Store the sample at -20 °C until HPLC analysis

Notes

Due to main limitations of Whole Culture Approach—namely, it is more laborious and does not result in monophosphate quantification—we have started to prefer Harvesting by Filtration Approach. Whole Culture Approach, however, stays the least manipulative approach for sampling and serves as a useful validation for the Filtration Approach. Furthermore, if there are problems with interfering peaks on HPLC, filtration derived samples can be refined either by the FPLC together with ethanol precipitation (for example, see **Supplementary Figure 10a**) or by the ethanol precipitation alone.

Nucleotide Samples for HPLC—Harvest by Filtration Approach

Materials

(1) Freeze-drier

Manifold freeze-drier with a decent cooling body so that it can handle vapours of organic acids

(2) Vacuum filtration system

Fast disassembly and filter retrieval is desirable. We use the one from DHI Laboratory Products:

http://c14.dhigroup.com/productdescriptions/filtrationequipment

(3) 45 µm, d=25 mm cellulose acetate filters from Sartorius (# 11106--25------N) or Whatman Protran BA (# 7000-0002).

Millipore HAWP02500 works also fine but, though not rigorously tested, might result in slightly weaker nucleotide signal. 5 mg bacteria per 25 mm filter is fine without clogging, for larger amounts, go for larger filters or several 25 mm filters in parallel. Some bacteria excrete sticky extracellular material and/or are slimy (e.g A. baumannii, B. thetaiotaomicron), then, more filter surface per 5 mg of bacteria is appropriate.

(4) 1 M acetic acid

For E. coli, acetic acid gives better stronger signal than formic acid for most of the nucleotides. Might though be different for other bacterial species.

(5) Vortex

(6) Tweezers for handling the filters

(7) Liquid nitrogen

(8) Serological pipettes (10 mL)

Sample acquisition

(1) Filtrate 10 mL of cell culture

Most critical step of sample acquisition is right after the liquids of culture medium have ran through the filter: filter should then be submerged into cold acid as fast as possible (see next step). 5-10 sec delays may easily alter nucleotide levels, at least for fast growing bacteria.

Use about 10-13 mL of E. coli culture of OD_{600} *0.5, however, for fast exponentially growing unstressed cells that much of a starting material gives small peak, so larger*

culture volumes might be necessary (30-40 mL). In that case, use of larger filters or several 25 mm filters in parallel is appropriate.

(2) Immerse filter in 600 µL ice-cold 1 M acetic acid in 1.5 mL tube

(3) Vortex 3-5 sec

(4) Quick freeze in liquid nitrogen, store at -80°C

Nucleotide extraction

(1) Thaw the sample on ice (will take 30-60 min)

Do not let sample to warm up, phosphoanhydride bonds of nucleotides are stable in acid only when kept cold! Multiple freeze-thaw cycles are not necessary: (1) there will be a second freezing in any case, before freeze-drying; (2) additional freeze-thaw cycles do not increase the signal in case of our protocol, if anything, the triphosphate levels go even lower upon extra freeze-thaw (data not shown).

(2) Extraction on ice for 30 min

After sample has thawed on ice, start the extraction with vortexing and continue with occasional vortexing

(3) Remove the filter:

(3.1) Invert the tube and tap the liquid onto the lid of the tube. Make sure the lid does not come open!

(3.2) Puncture the bottom of the tube with a hot syringe-needle (heated in the flame of alcohol or gas burner) and insert the 1.5 mL tube into 2 mL tube

(3.3) Centrifuge tubes briefly (up to 8,000 G) to get the liquid phase of the sample into the 2 mL tube

Work in batch of 6-8 tubes in parallel, otherwise, sample might warm up during handling. In rotor, leave at least one empty position between each tube, otherwise lids of tubes will intertwine and might break off.

(3.4) Optional: if sample is bigger than 600 µL or if the freeze-drier is not that good keeping the samples frozen, suspend the sample in 2 mL tube and transfer to 15 mL tube for freeze-drying

(4) Freeze-drying

(4.1) Freeze the samples in liquid nitrogen

(4.2) Put a parafilm on tube, puncture the parafilm with needle

(4.3) Put the tube into a tube holder of some sort [this step is optional]

(4.4) Freeze in liquid nitrogen again (to keep it frozen)

(4.5) Take samples to freeze-drier

Takes about 6 h to dry (time needed depends a lot on the freeze-drier). O/N is also an option but then one has to be more rigorous when taking the sample up in water

(5) Dissolve in 200 µL ice-cold mQ

(6) Spin at maximum speed for at least 30 min, collect clear supernatant

(7) Store at -20 °C until analysis on HPLC

Strong Anion Exchange HPLC

Materials

(1) System:	Agilent 1100 with thermostated autosampler, column oven and DAD detector
(2) Column:	SAX 5 µm 4.6x150 mm, either Sphereclone (Phenomenex # 00F-4149-E0) or Spherisorb (Waters # PSS832713) *Looking at the literature, Partisil 10 µm 4.6 x 250 mm (Whatman) is also a common choice. It might last longer than the columns we are using ((Pogolotti and Santi 1982), personal communication with Andrei Chabes).*
(3) Pre-column:	SecurityGuard cartridges (Phenomenex # AJ0-4311) in a holder (Phenomenex # KJ0-4282).
(4) $NH_4H_2PO_4$	HPLC grade (Fluka # 17842)
(5) Nylon filters:	0.2 µm 47 mm (Sartorius # 25006--47------N or Whatman # WHA7402004)

General notes

(1) Column temp 26 °C, injection volume most often 100 µL (equivalent of 2.5 ODU of bacterial culture e.g. 5 mL of OD_{600} 0.5. In unstressed conditions of fast growing bacteria, to detect ppGpp, be prepared to use 3-5 fold more material)

(2) For column storage and wash, methanol does a better job than acetonitrile. Methanol is more efficient in cleansing of the column from charged compounds. Better cleansing results in faster outset and therefore shorter column uptime during the next session. We did not try phosphoric acid wash that is occasionally recommended (e.g. (Pogolotti and Santi 1982)).

(3) The main complication is the rapid deterioration of retention times (could be assigned to the complex, crude samples we usually have and very salty buffers) and short lifespan (only about 200-250 runs). Retention times decrease at different speed for nucleotides of interest and interfering peaks, making gradient program especially tricky to adjust and maintain. We have tried different column regeneration techniques but they seem to result in even faster wear off of the column. Retention decreases gradually during runs, however, expect to see more considerable decrease right after each storage of column, therefore, having several samples in one longer session is recommended.

(4) One has to remain careful when using high salt HPLC buffers. First, if the instrument is not cleaned properly, the remains of the salt can form crystals in pumps and seals; thus, using a pump seal wash system is recommended. Second, organic solvent for column storage or wash should be pumped through the system only after a thorough wash with water in order to avoid precipitation of buffer salts.

Gradient program

Buffers: A – 0.05M $NH_4H_2PO_4$ pH 3.4;

B – 0.5M NH_4H_2PO4 pH 3.4

Make buffer B (27.3 g/L, adjust pH with H_3PO_4, bring volume to 1L), filtrate (0.2 µm 47 mm nylon), and then use fraction of it to make buffer A by just diluting in mQ, no filtration nor pH adjustment of fresh buffer B. Store the phosphate buffers at 4 °C when not in use and re-filter every couple of days before use. Make buffers from HPLC grade reagent, otherwise baseline deviation and interfering peaks might be a problem. Most probably, HPLC grade KH_2PO_4 can be used instead of $NH_4H_2PO_4$.

Usually the program for a new column is the following:

	%B	flow rate (m/min)
0 min	0	1
30 min	100	1

45 min	100	1

Notes on gradient program

(1) We have tried to improve the resolution (initial isocratic, stepwise gradients etc) but the linear gradient is the best, by a large margin.

(2) As column gets older, retention decreases. Therefore, decrease the final percentage of B (about 20% steps are appropriate: 100%, 80%, 60%, 40% and then the column is finished) and include short wash with 100% buffer B at the end of each run. If possible, consider mobile phase conditioning pre-columns as it is recommended for Partisil SAX (the one for Partisil is called Solvecon Guard Column), it helps to saturate the mobile phase with silica and helps to minimize column dissolution.

(3) Painstakingly, as the column gets older, retention decreases at different rate for different peaks—for peaks of interest and for interfering peaks—so that on top of adjusting buffer strength and/or gradient, one has to deal with certain ranges of runs in column lifespan during which one or the other nucleotide is nevertheless not resolved

(4) We do realize the adjustments in buffer strength are tedious and obscure. We therefore describe now a typical gradient run session which consists of following steps. First, the storage solution i.e. methanol is washed off with water. Second, the column is cleaned with high salt buffer by means of alternating 2-4 times between buffers A (low salt) and B (high salt) until the UV detection trace becomes reproducible. Third, the column is equilibrated with buffer A, followed by injection of the first sample. The first run is followed by an injection of the same sample spiked with nucleotide standards in in order to determine the identities of peaks. Fourth, sequence of samples is run and every 10th-20th sample is run twice, first without and then with the nucleotide standards spiked in. Finally, immediately after the session, salt is washed off the column with water and the column is stored in 100% methanol.

(5) Regarding the poor resolution of ATP in some early runs. What exactly seems to happen is that some peaks—with a retention time between ATP and GTP (where dATP and UTP are to be expected)—over the course of runs, due to change in retention time, re-positions in front of ATP. Comparison to the chromatograms reported by Buckstein *et al.* suggests that part of it might be CTP. If the interfering peaks were UTP, CTP, dNTPs, NAD(P)H degradation and/or some other negatively charged UV-absorbing substances, we did not pursue to find out, however, the problem was more pronounced for *E. coli*

samples than for *B. subtilis* (although the difference could have been due to somewhat different media used for the species, *B. subtilis* was grown as previously described (Kudrin, Varik et al. 2017)).

(6) As mentioned also in main text, increasing the resolution by using a longer column is likely to fail due to significant widening of the peaks (**Supplementary Figure 1b**). The widening, however, cannot be easily counteracted by increasing ionic strength of the buffer because of the already high salt concentrations necessary to elute the nucleotides from a shorter column.

Isocratic program

Buffer: 0.36M $NH_4H_2PO_4$, pH 3.4, 2.5% (v/v) acetonitrile

39.3 g/L, adjust pH with H_3PO_4, add acetonitrile, and bring volume to 1L. Filtrate (0.2 μm 47 mm nylon). See notes for handling of phosphate buffers in the section of a gradient program above.

Program: Flow rate 2 mL/min, temperature 26°C

Notes on isocratic program.

(1) Isocratic program is what we use nowadays almost exclusively as opposed to gradients. The routine is to measure ppGpp and ppGpp on SAX and quantify the rest of the nucleotides on Ion-Paired Reverse Phase

(2) Most optimal for ppGpp measurements is to keep ppGpp retention time between 7.5 to 15 min. Be alert to point any interfering peaks, though, and if possible, verify the identity of a spectrum of a guanosine base.

(3) As the retention time decreases, come down with a flow rate—1.5 mL/min and 1 mL/min could be tried—and if that is not sufficient, come down with the buffer concentration, about 25% step is appropriate: 0.27M (29.5g/L), 0.19M (20.8g/L).

(4) We have not tested, but with a fresh column, instead of a 0.36M at 2 mL/min, one could try 0.5M at 1.5 mL/min or even at 1 mL/min.

Ion-Paired Reverse Phase HPLC

Column: Kinetex C18 4.6×150 mm 2.6 µm (Phenomenex # 00F-4462-E0)

*We have used Waters Symmetry C18 4.6×150 mm 3.5 µm, and it works well (**Supplementary Figures 2** and **3**), however, gradient needs to be twice as long to give about the same resolution as with Kinetex. Also, the sensitivity is lower with Symmetry.*

Precolumn: SecurityGuard ULTRA (# AJ0-9000)
with appropriate cartridges (# AJ0-8768)

Buffers: A: 5 mM TBA-OH (Sigma # 86854), 30 mM KH_2PO_4 (Fluka # 60221),
pH 6.0 (with H_3PO_4)
B: acetonitrile (Sigma # 34851)

Gradient: 0-20 min linear gradient 5-35% acetonitrile 0.8 mL/min 26°C
stop at 25 min
equilibration for 15 min

Injection: 30-50 µL (approximately 1.25 ODU of bacterial culture e.g.
2.5 mL of OD_{600} 0.5)

Preparation of buffers. To make 1L of buffer A, dissolve 3.24 g 40% t-butylammoniumhydroxide (TBA-OH) in ~800 mL mQ. Add 4.08 g KH_2PO_4 and adjust pH to 6.0 with H_3PO_4. Finally, bring the volume to 1L with mQ and filtrate through 0.2 µm nylon filter. Store the buffers at 4 °C when not in use and re-filter every couple of days before use.

Wash with EDTA instead of including it in buffer or in sample. Regardless if it is due to two-valent cations or something else, column needs periodic wash with EDTA (**Supplementary Figure 2d, 2e** and **2f**), otherwise the shape of the peak of highly phosphorylated nucleotides deteriorates. Note that EDTA has no effect on fresh column (**Supplementary Figure 2c**). Every time we start HPLC, we thus perform a blank gradient run (in case of Waters Symmetry column, we performed a shortened 20 min gradient) in which we inject 100 µL 50 mM EDTA pH 8.0. Cserjan-Puschmann and colleagues recommend to include EDTA in buffer at 50 mM (Cserjan-Puschmann, Kramer et al. 1999). We find it to result in a baseline protrusion (a very wide and non-symmetric peak) in the middle of the gradient that (i) is only somewhat reproducible in size and

location, therefore hampers the quantification of the nucleotides, (ii) does not disappear even at IPTG concentrations as low as 1 μM. We therefore resorted to EDTA injection-wash. Likewise, if contemplating to increase the stability of nucleotides via addition of EDTA during sample processing (Lundin and Thore 1975), retain that it might interfere with the HPLC (in fact, we had similar problems with EDTA on gradient SAX-HPLC).

Shutting down Kinetex Core-Shell C18 column. In our experience, one needs to perform shutdown of Kinetex Core-Shell C18 column with some caution, if higher than 65% ACN is to be used for storage. We did not investigate it thoroughly but it seems as if something is crashing out—probably ion-pair— and blocking the pre-column although the system had always been first washed with water. If such a blockage happens, changing the pre-column cartridge will restore the performance. We often stored the column in 65% ACN, however, favoured storage in 100% ACN and for the latter, we ran 5% ACN in water for 20 min at 0.8 mL/min (about 10 column volumes) to get rid of buffer salts, then brought flow rate to 0.4 mL min and switched to 65% ACN which we ran for 5 minutes. Finally, a linear gradient was ran from 65% ACN to 100% ACN in 15 minutes, after which, column was ready to be shut down.

General notes. As with SAX columns, we do get only 200-300 runs out of a C18 column in IPRP mode. That is, however frustrating, to be expected in case of complex biological material (Snyder, Kirkland et al. 1997). We tried some thorough wash regimes recommended by manufacturer but if anything, it resulted in faster wear-down of the column. We did not try wash with DMSO and with acetic acid (Gebelein, Merdes et al. 1992). Therefore, the only maintenance we do is regular replacement of pre-column, careful storage in solutions high in acetonitrile and EDTA injection wash.

We did attempt to use the IPRP-HPLC program as described by Buckstein and colleagues (Buckstein, He et al. 2008), however, we used HPLC-grade t-butyl ammonium phosphate instead of PIC A. For the majority of nucleotides, there was a massive increase in the baseline with the signal coming, most likely, from the eluting ion-pairing agent itself, or from impurities it contains. We therefore suggest either to use our protocol (which essentially adopts the one from Payne and Ames (Payne and Ames 1982)) or to follow the Buckstein protocol to the dot, including the use of PIC A.

Although dNTPs are probably not interfering with NTP measurements (dNTPs are 1-2 orders of magnitude less abundant (Nick McElhinny, Watts et al. 2010)) and quantification of dNTPs is out of the scope of this work, both isocratic and shallow gradient IPRP-HPLC hold the promise to skip the NTP removal step—which is usually done via periodate-methylamine oxidation (Garrett and Santi 1979, Tanaka, Yoshioka et al. 1984, Harmenberg, Cox et al. 1990) and/or boronate affinity chromatography (Payne and Ames 1982)—to resolve both dNTPs and NTPs in one run (**Supplementary Figure 3b** and **3c**; see also (Arezzo 1987, Cross, Miller et al. 1993, Di Pierro, Tavazzi et al. 1995)).

Settings of Diode Array Detector

Response-time	0.2 sec
UV trace recorded:	252 nm, bandwidth 4, reference off, slit 4 nm
Spectrum, if acquired:	
range	200 to 400 nm
step	2 nm

Integration parameters for Agilent ChemStation (Software Version: Rev. B.04.03-SP1). Manual integration was avoided (used no more than for about one peak in 5-10% of the HPLC traces). Note that overlapping peaks, in case of a diode array detector, can be accurately quantified as discussed by Cross et al (Cross, Miller et al. 1993).

Initial Events For All Signals

Tangent Skip Mode	New Exponential
Tail Peak Skim Height Ratio	1.00 (6.00, 2.00)
Front Peak Skim Height Ratio	6.00 (1.00, 2.00)
Skim Valley Ratio	2.00 (20.00)
Baseline Correction	Advanced
Peak to Valley Ratio	500

Specific Events For Signal

Slope Sensitivity	10 [2, 5, 20, 50, 100]
Peak Width	0.01 (adjusted to narrowest peak of interest)
Area Reject	5 (or, use common sense)

Height Reject	4 (or, use common sense)
Shoulders	OFF

Bacterial Cell Number and Volume Estimations

Cell dimensions, although apparently trivial, are hard to measure with great precision because of unavoidable technical hardships. Therefore, what we have attempted here is only an approximation of the cell volume. Cultures were grown in defined minimal medium (MOPS 0.4% glucose at 37 °C with vigorous aeration) (**Supplementary Figure 7a**). Samples were drawn and stained with nigrosin (mixed with 1% nigrosin, spread onto microscope slide and air dried (**Supplementary Figure 7b**)) and microscopy images were analyzed by ImageJ (NIH), pixel-to-μm conversion was achieved by comparison with commercial beads. About 100-150 cells were measured for each time point. As described by Fry (Fry 1990), width (W) and length (L) were derived from perimeter (P) and area (A):

$$W = \frac{P - \sqrt{P^2 - 4\pi A}}{\pi}$$

$$L = \frac{P}{2} + W(1 - \frac{\pi}{2})$$

Then, volume (V) was calculated approximating all bacteria to be a cylinder with two hemispherical caps:

$$V = \frac{\pi}{4} W^2 (L - \frac{W}{3})$$

Finally, it was assumed that the periplasm accounts for a 0.21 fraction of cell volume (Stock, Rauch et al. 1977).

The cell volume decreases from 1.2±0.3 in exponential phase to 0.5±0.2 femtoliters in stationary phase (**Supplementary Figure 7c and 7d and Supplementary Table 1**)

Data Handling and Analysis

All the reported mean values and descriptors of spread are geometric unless indicated with '±' sign, in which case, arithmetic counterparts were used. Variation or uncertainty was expressed mostly with 95% confidence intervals; on graphs, however, we used standard error of the mean to avoid clutter in some cases and to keep it uniform in others. Generally, outlier removal was avoided but when it was applied, Tukey fences (measurements deviating more than 1.5-times the interquartile range) were used on logarithmically transformed data (outliers did not account for more than about 2-3 % of the data).

SUPPLEMENTARY FIGURES

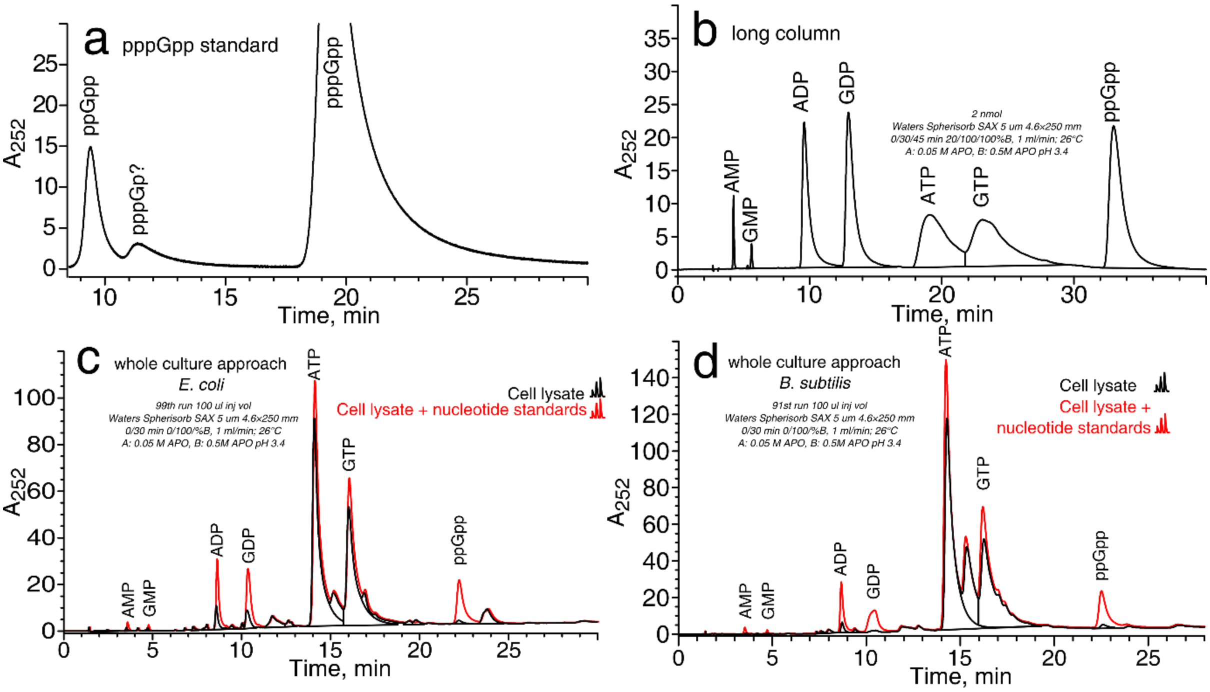

Supplementary Figure 1 | Examples of performance of Strong Anion Exchange.

(a) Isocratic SAX-HPLC, with a program and column as described on **Figure 2**, was used to determine the retention time and absorbance spectrum of a pppGpp standard. The presence of degradation products—ppGpp, and most likely pppGpp—was apparent. **(b)** In an attempt to improve the resolution of gradient SAX-HPLC, we switched from a column length of 150 to 250 mm. As anticipated, this results in increased retention times and slightly improved resolution. All the gain in resolution, however, was negated by considerable widening of the peaks. What is shown here is our attempt to alleviate the widening, we increased salt concentration of the program to decrease retention times. Nevertheless, comparison with **Figure 3a** makes obvious that longer column does not give advantage in terms of resolution. **(c-d)** Column chromatography and LiCl precipitation refinement inherent to our whole culture approach do get rid of many peaks (compare with **Figure 3b**) but the resulting signal is weak and close to the background for mono- and diphosphates of a cell lysate from *E. coli* (c) and from *B. subtilis* (d).

HPLC conditions: (a) SphereClone column 5 µm 4.6 × 150 mm was run with buffer containing 0.36 M $NH_4H_2PO_4$ pH 3.4, 2.5% acetonitrile at 26 °C with a flow rate of 1.5 mL/min. (b) Spherisorb 5 µm 4.6×150 mm column was run at 1 mL/min, 26 °C. Buffer A: 0.05 M $NH_4H_2PO_4$, pH 3.4. Buffer B: 0.5 $NH_4H_2PO_4$, pH 3.4. Gradient: 20/100/100 %B at 0/30/45 min. (c-d) Same as in (b), except the gradient: 0/100/100 %B at 0/30/45 min.

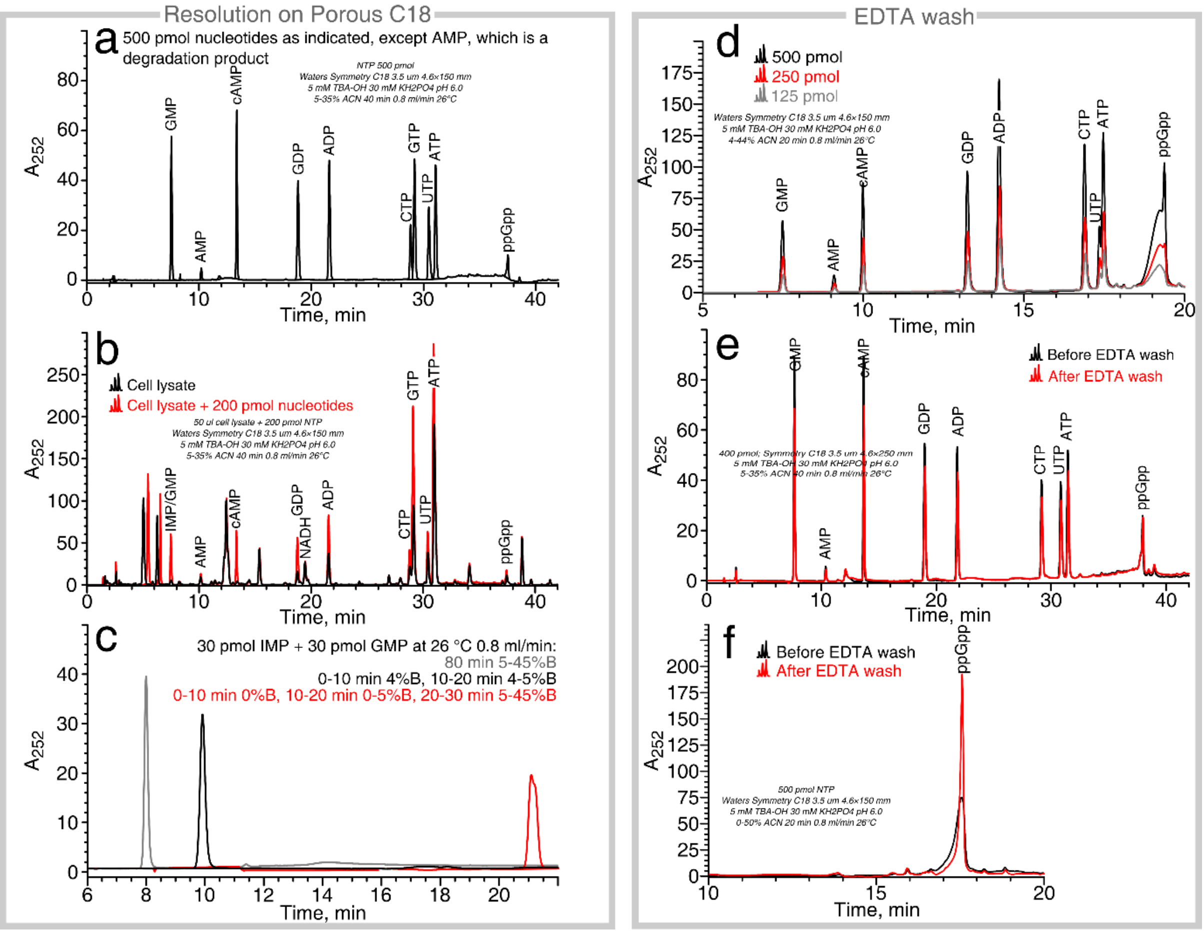

Supplementary Figure 2 | Resolution of nucleotides, except IMP-GMP, is good on conventional porous particle C18 column in IPRP mode; peak shape of ppGpp, however, deteriorates as column gets older and this can be counteracted by EDTA wash.

(a) Nucleotides in buffer and **(b)** in cell lysate are well resolved also on conventional C18 column with porous particles (see also **Supplementary Figure 3** for representative chromatograms). Sharing the property with pellicular columns, however, IPRP with the current buffer system does not separate GMP from IMP. **(c)** Trying various gradient programs makes clear that separation of IMP and GMP can not be achieved this way. If separation of the IMP and GMP is a necessity, consider buffers of lower pH and consult Mack *et al* (Mack, Reed et al. 1985). **(d)** After about 50 runs, in spite of few times of wash-and-storage in high levels of acetonitrile, ppGpp peak starts to deform. Unfortunately, at lower quantities of ppGpp, it is the narrow part of the peak that disappears and what left is poorly resolved and probably insufficiently quantifiable 'hump'. We speculated that it is the accumulation of divalent cations and tried to wash with EDTA injections (100 µl 50 mM pH 8.0). **(e)** EDTA wash has negligible effect on a column with less than 50 runs,

whereas **(f)** the wash imposes substantial effect on the shape of a ppGpp peak improving sensitivity, resolution and robustness.

HPLC conditions: Waters Symmetry C18 3.5 µm 4.6×150 mm; 0.8 ml/min, 26°C. A: 5 mM TBA-OH, 30 mM KH_2PO_4, pH 6.0; B: ACN. Gradients as indicated on panels.

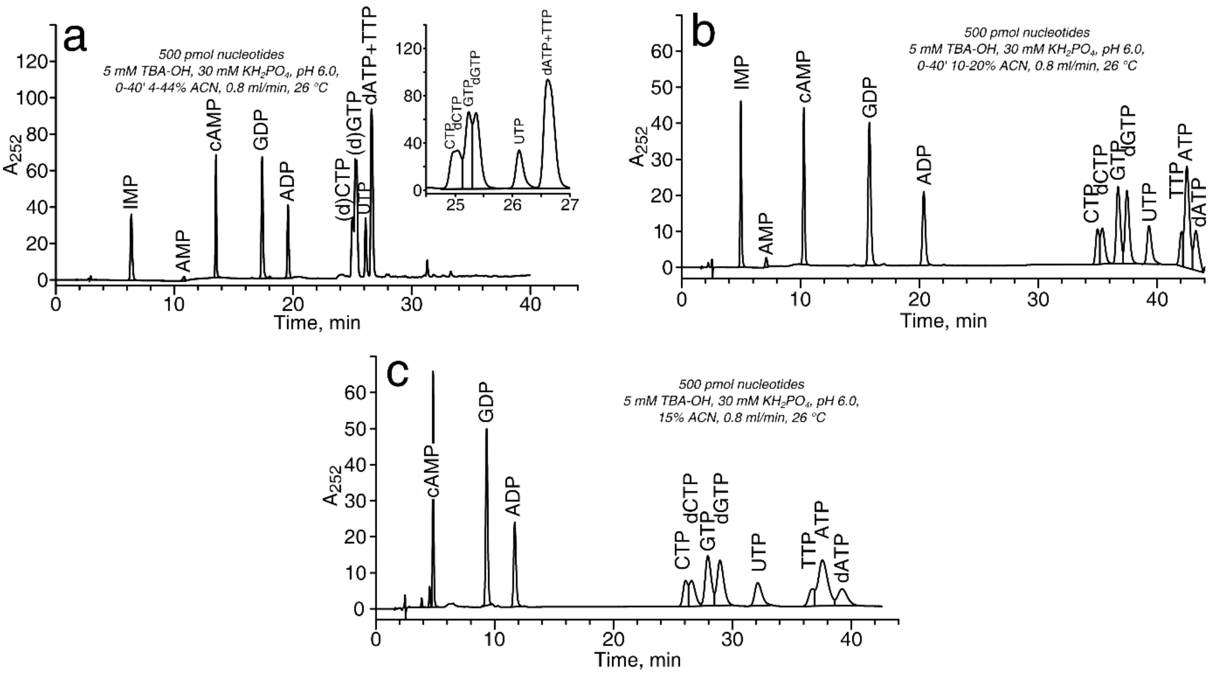

Supplementary Figure 3 | dNTPs could be resolved from NTPs by shallower gradient or isocratic IPRP.

(a) dNTPs were not separable from corresponding NTPs with our regular gradient IPRP program (4-44% B in 40 min). Subset shows close-up view of (d)NTP region if the chromatogram. **(b)** Our best result with a shallow gradient regime (10-20% B in 40 min). **(c)** Resulting chromatogram of an isocratic regime (15% B).

HPLC conditions: 500 pmol of each nucleotide standard as indicated (except AMP which was a degradation product). Waters Symmetry C18 3.5 µm 4.6×150 mm; 0.8 ml/min, 26°C. A: 5 mM TBA-OH, 30 mM KH_2PO_4, pH 6.0; B: ACN

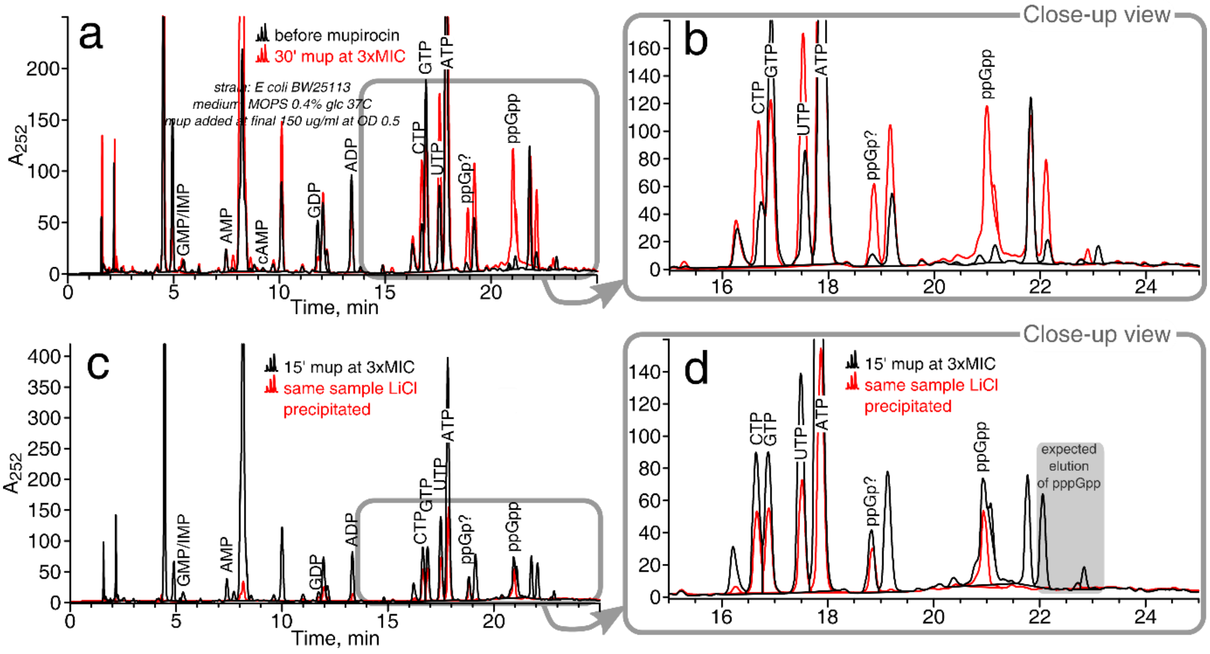

Supplementary Figure 4 | Using IPRP-HPLC with UV detection, ppGpp is undetectable in rapidly growing cells and pppGpp cannot be measured even from cells going through stringent response.

(a) *E. coli* culture was grown in defined minimal medium (MOPS 0.4% glc at 37 °C with vigorous aeration) until OD_{600} 0.5 and stringent response was induced by 150 µg/mL of mupirocin (3×MIC). Using rapid filtration, samples were taken for nucleotide measurements before (black trace) and after 30 minutes of mupirocin addition (red trace). **(b)** Close up view of (a) to zoom in on ppGpp in unstressed cells. **(c)** *E. coli* cells were harvested by filtration after 15 minutes of induction of stringent response by mupirocin as described above. The final sample, taken up in water, was split into two, one of which was subjected through LiCl precipitation (red trace) and the other one served as untreated control (black trace). LiCl precipitation retains most of the highly phosphorylated nucleotides—including the one which we speculate to be ppGp, and ppGpp—but there is no signal of pppGpp. **(d)** Close up view of (c) to zoom in on highly phosphorylated guanosines.

HPLC conditions: Kinetix C18 2.6 µm 4.6×150 mm, 26 °C. Buffer A: 5 mM TBA-OH, 30 mM KH_2PO_4 pH 6.0. Buffer B: 100% acetonitrile. Gradient: 0-20 min 5-35% B.

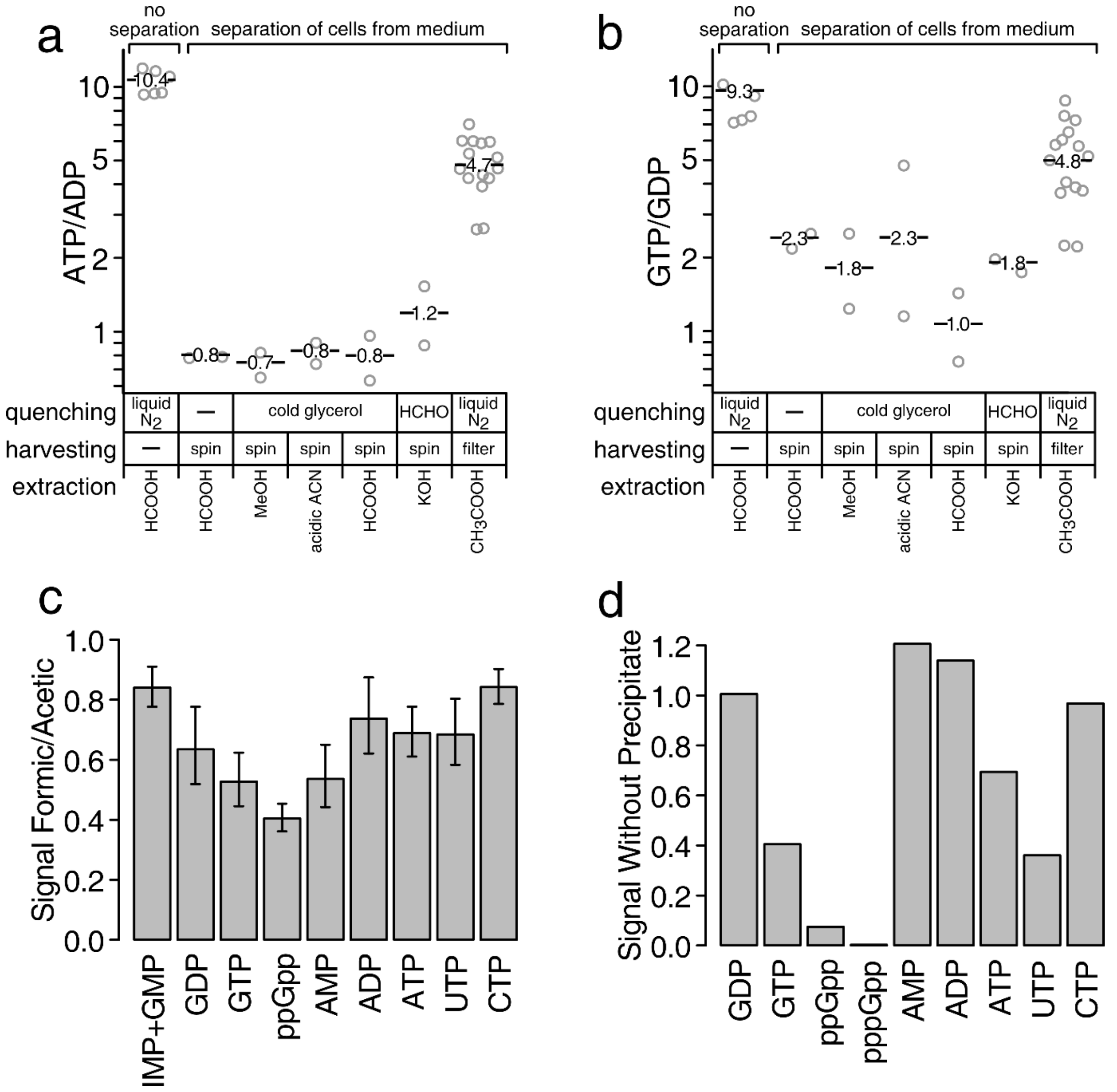

Supplementary Figure 5 | Different approaches to sample preparation.

(a) Based on ATP/ADP ratio of rapidly growing *E. coli* cells, filtration is the only form of harvesting that gets close to the ratios observed with least manipulative sampling i.e. no harvesting. Furthermore, centrifugation appears very inappropriate for harvesting even if preceded by quenching in cold glycerol or by aldehyde fixation. In general, ATP/ADP ratios way below 5 should be considered with caution as discussed by Pogolotti *et al* (Pogolotti and Santi 1982). **(b)** Similar pattern as for ATP/ADP, although less pronounced, was observed also for GTP/GDP ratios. **(c)** Acetic acid extraction of filtered cells gives stronger signal than formic acid. We speculate it is due to better compatibility with freeze-drying. Results are expressed as mean from two biological replicates with three technical replicates each. Error bars stand for s.e.m. **(d)** In case of acetic acid

extraction of filtered cells, release of nucleotides from cells seems to become final during the freeze-drying step. Removal of acid precipitated material from sample prior freeze-drying has resulted in weaker signal also elsewhere (Nazar, Lawford et al. 1970).

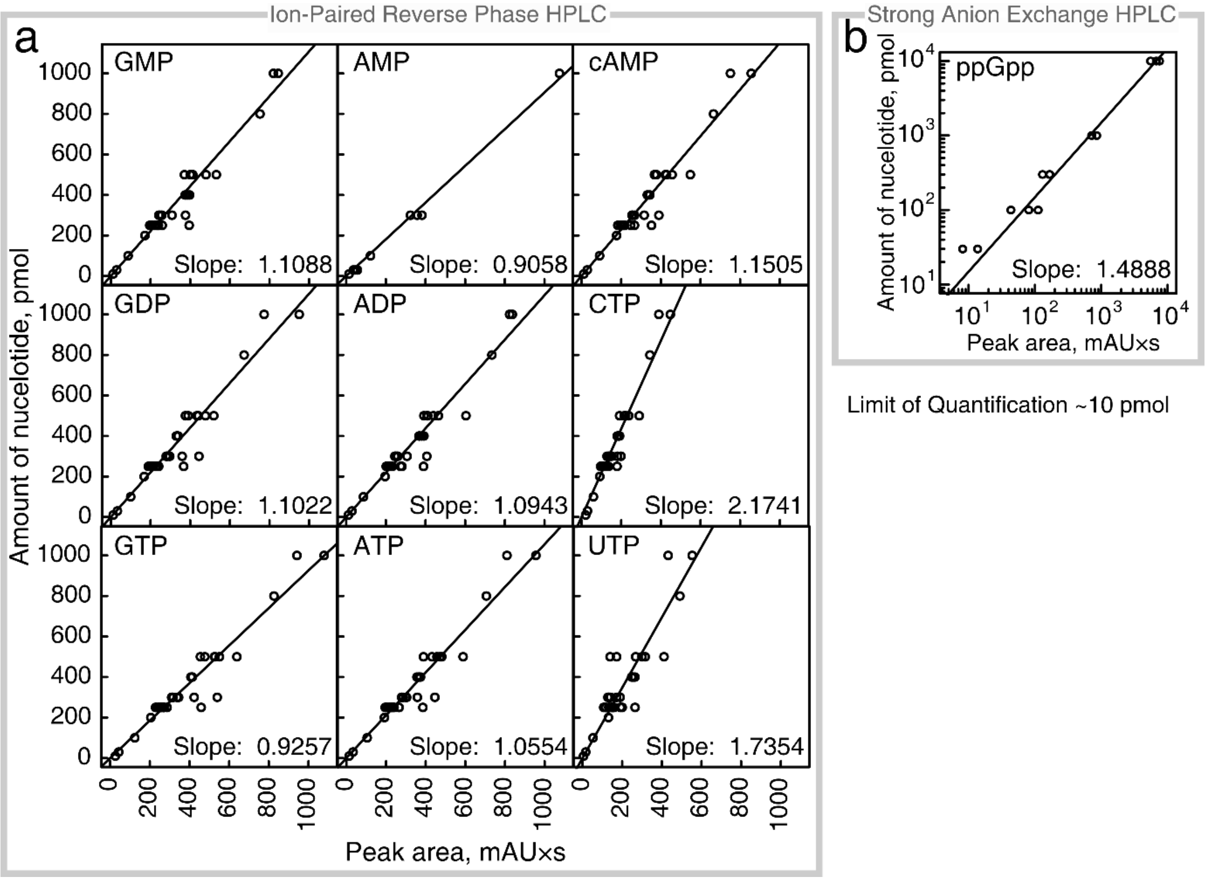

Supplementary Figure 6 | Calibration curves of nucleotides.

Known amounts of nucleotides were resolved by **(a)** IPRP-HPLC (GMP, AMP, cAMP, GDP, ADP, CTP, GTP, ATP, UTP) or by **(b)** SAX-HPLC (ppGpp). Resulting peaks of chromatograms were integrated and areas plotted against the amount of nucleotide standard injected. As it is obvious from (b), HPLC with a DAD detector gave an excellent dynamic range for at least four orders of magnitude. Limit of quantification, defined as signal/noise ratio of >10, was about 10 pmol for all nucleotides.

HPLC conditions: (a) IPRP-HPLC, Kinetix C18 2.6 µm 4.6×150 mm, 26 °C. Buffer A: 5 mM TBA-OH, 30 mM KH_2PO_4 pH 6.0. Buffer B: 100% acetonitrile. Gradient: 0-20 min 5-35% B. (b) isocratic SAX-HPLC: SphereClone column 5 µm 4.6×150 mm, $NH_4H_2PO_4$ pH 3.4 with an appropriate ionic strength (0.36M, 0.27M or 0.19M), 2.5% acetonitrile at 26 °C with an appropriate flow rate (0.5-1.5 mL/min). For what is considered appropriate, see Supplementary materials and methods on isocratic SAX-HPLC.

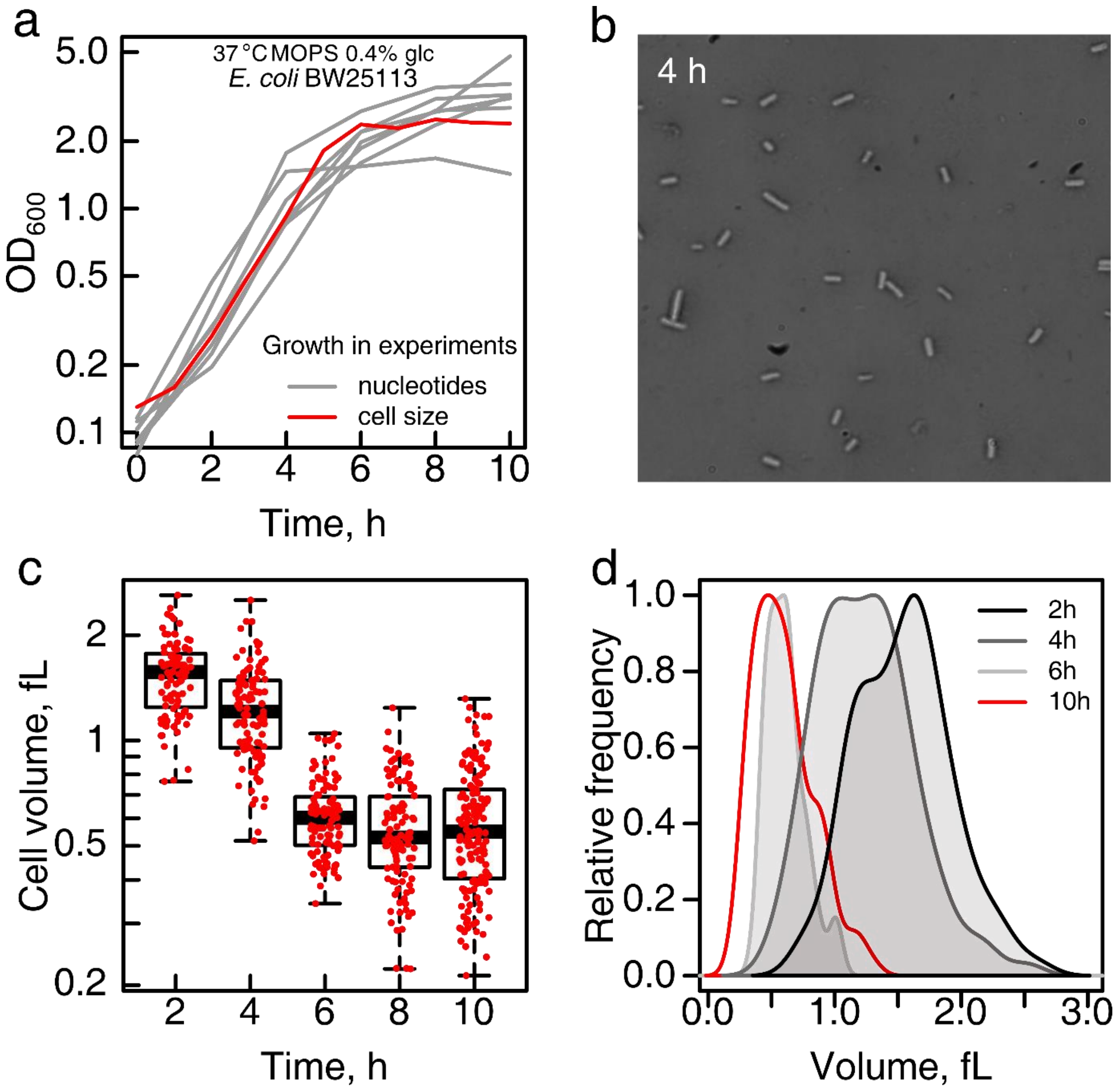

Supplementary Figure 7 | *E. coli* cell size distribution during the growth in defined glucose minimal medium.

(a) *E. coli* cultures were started at OD_{600} 0.1 in defined minimal medium (MOPS 0.4% glucose at 37 °C with vigorous aeration) and grown into stationary phase. Growth was followed by OD_{600} and resulting traces are shown for the experiments were samples were taken for nucleotide measurements (grey) and for the single experiment when cell concentration and dimensions were measured (red). **(b)** For cell dimension measurements, samples were removed at 2, 4, 6, 8, 10 hours of growth and cells were stained with 1% nigrosin, spread onto microscope slide, air dried and imaged using light microscope. A representative microscope image is shown from 4 h timepoint. **(c)** For each timepoint, cell volumes were calculated for 88-153 cells as described in Supplementary

Materials and plotted here with box-and-whisker blots with the whiskers standing for range. For exact numerical values of mean and standard deviation, consult Supplementary Table 1. **(d)** Same as (c) except that cell volume distribution is expressed as density for better visualization of the distribution.

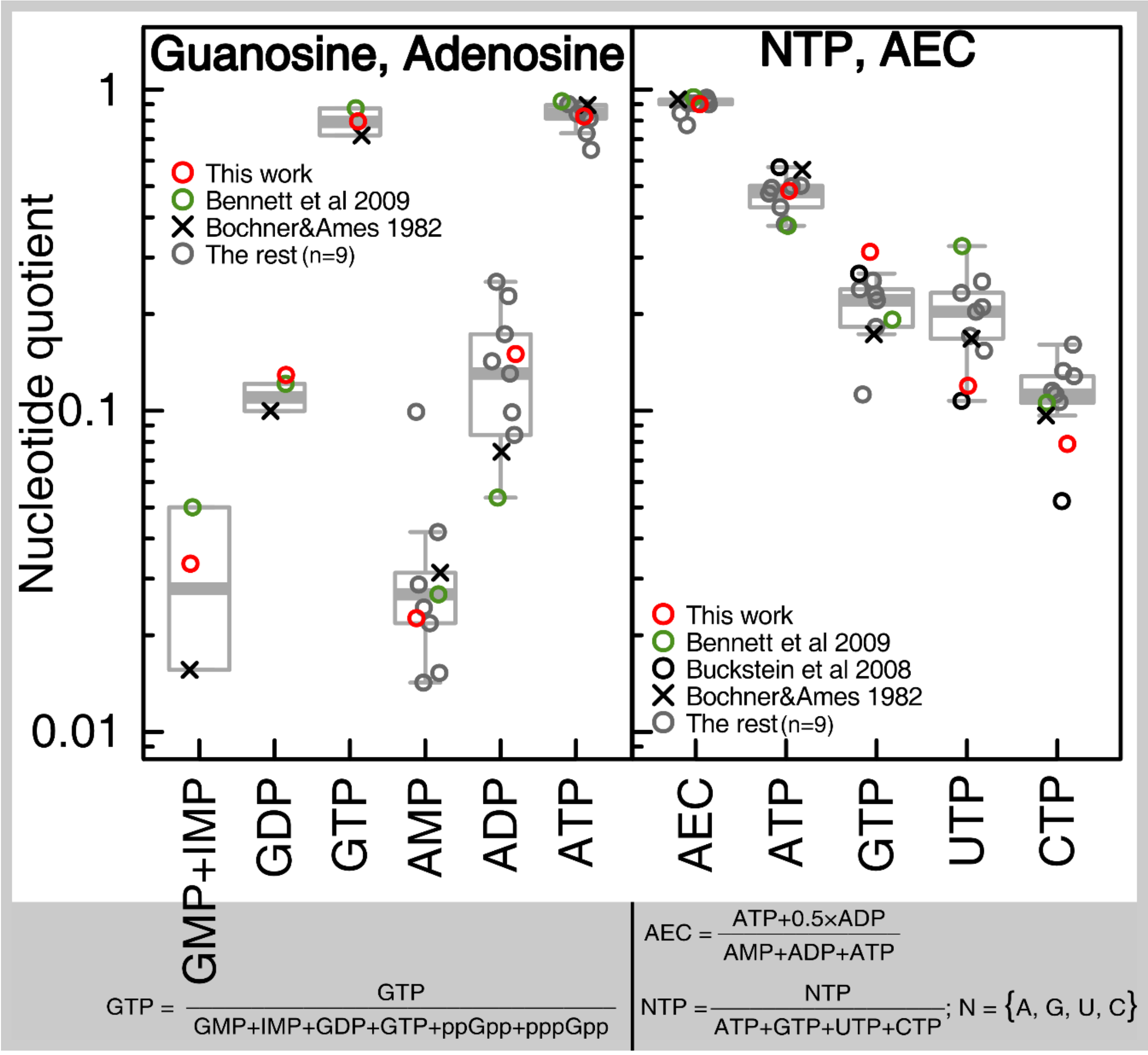

Supplementary Figure 8 | Reported nucleotide quotients of exponentially growing bacterial cells growing in defined medium agree well with our results.

The relative abundance of nucleotides was expressed as shown on inset and presented in comparison with our work (red circles). Instead of *E. coli*, Bochner and Ames (black crosses) measured the nucleotide levels in *Salmonella typhimurium*, however, their report has become the textbook reference of bacterial nucleotide levels (Bochner and Ames 1982). Work of Bennett *et al.* (green circles) is exceptionally methodologically rigorous and, besides nucleotides, covers a wide range of other metabolites (Bennett, Kimball et al. 2009). The methodology implemented by Buckstein *et al.* (black circles) is the closest to our work and they quantify almost the very same set of nucleotides during growth from exponential into stationary phase (Buckstein, He et al. 2008). The results of 9 other reports (grey circles, aggregated set) were pooled together for descriptive statistics of median and interquartile range as shown by boxplots (Franzen and Binkley

1961, Neuhard and Munch-Petersen 1966, Edlin and Neuhard 1967, Bagnara and Finch 1968, Lowry, Carter et al. 1971, Holms, Hamilton et al. 1972, Poulsen and Jensen 1987, Vogel, Pedersen et al. 1991, Taymaz-Nikerel, de Mey et al. 2009). The whiskers stand for range still within 1.5-times the interquartile range of lower/upper quartile. Note that guanosine ratios reported by Bennett *et al.* were calculated without taking into account the (p)ppGpp levels since in this report (p)ppGpp was not quantified and, therefore, not reported (Bennett, Kimball et al. 2009). We believe that this did not significantly affect the final result since the (p)ppGpp levels in rapidly growing cells are low and constitute only a small fraction of the total guanosine pool.

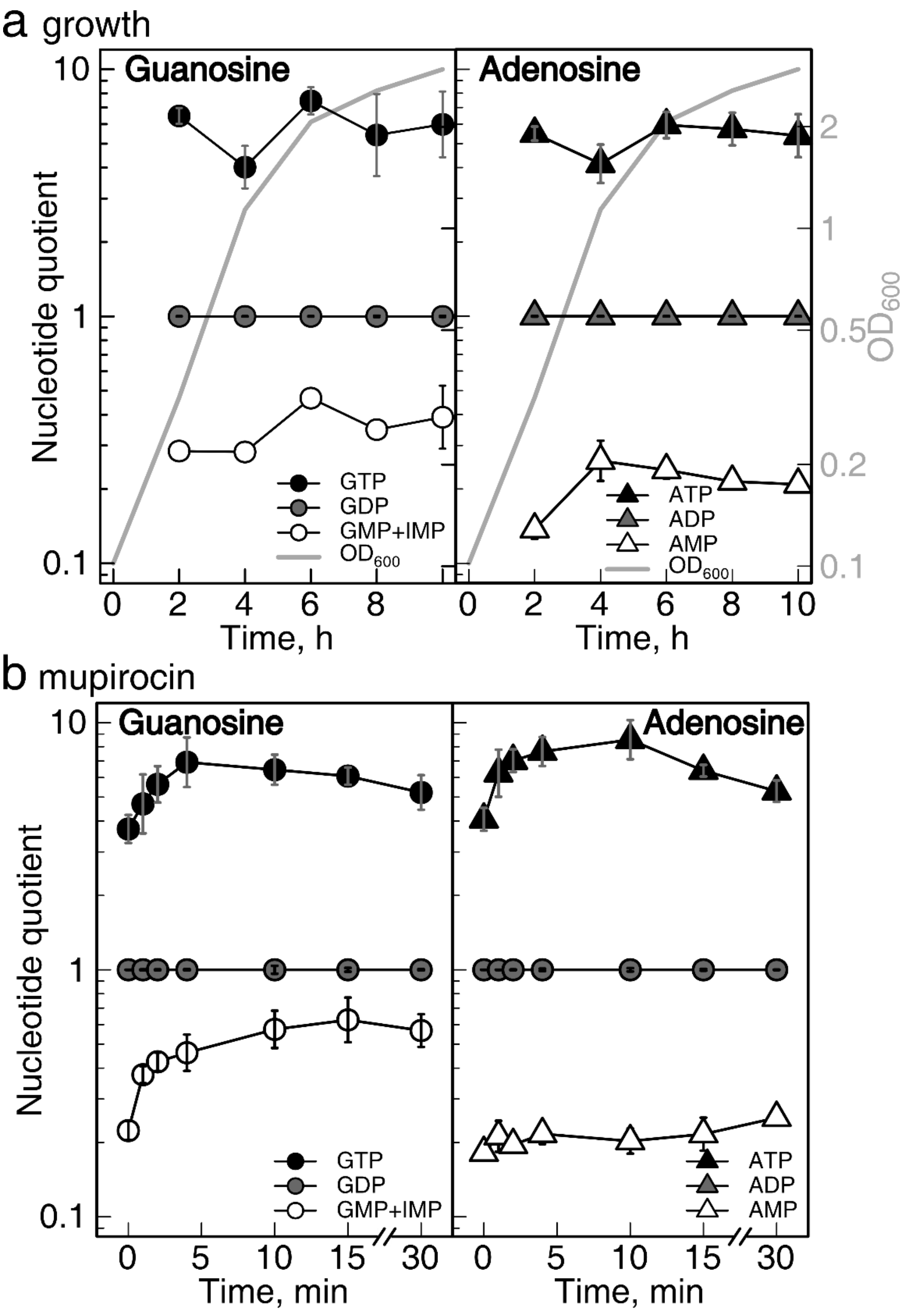

Supplementary Figure 9 | Ratios of NTP/NDP/NMP are kept stable throughout growth curve and do change temporarily less than 2-fold in case of acute stringent response.

(a) The abundance of intracellular nucleotides of *E. coli* is shown here in comparison to the levels of NDP (the figure is assembled form the same set of experimental data as on **Figure 4a**). Therefore, the abundance of either GDP (for guanosines) or ADP (for adenosines) is by definition 1. Cells were grown in defined minimal medium (MOPS 0.4%

glc at 37 °C with vigorous aeration), harvested by filtration, nucleotides extracted with acetic acid and quantified using gradient IPRP. GMP was not resolved from IMP. Error bars indicate standard error of the mean of biological replicates (n=7). **(b)** Cells were grown in the same conditions as described above until OD_{600} of 0.5. Next, stringent response was induced by mupirocin (3-times the MIC, 150 µg/ml) at time point zero. At times indicated, cells were harvested by filtration, nucleotides extracted with acetic acid and quantified using gradient IPRP. Here, the transient accumulation of GTP and ATP is apparent. Also, increase in GMP + IMP is probable, however, caution should be executed in the interpretation: given the excess of GTP, even slight changes in GTP degradation would result in large changes of GMP + IMP. Error bars indicate standard error of the mean of biological replicates (n=8 at zero time point, otherwise n=3-4).

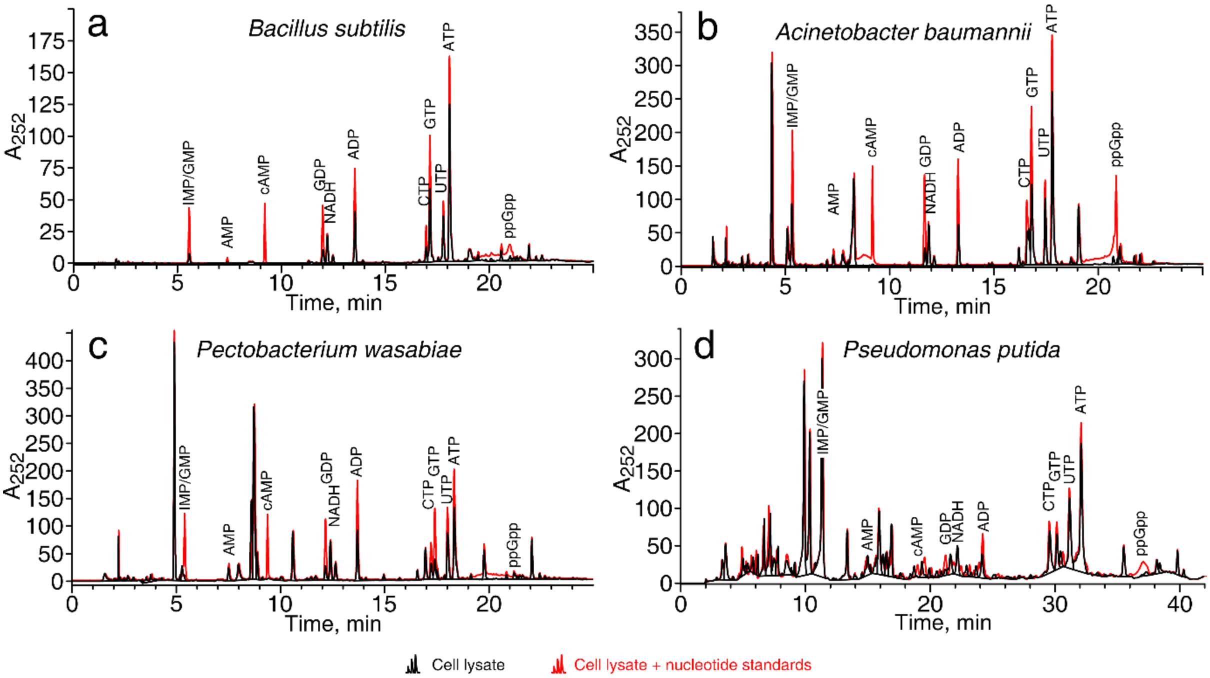

Supplementary Figure 10 | Filtration approach can be successfully applied to several bacterial species besides *E. coli*.

The chromatograms of intracellular nucleotides of *Bacillus subtilis* **(a)**, *Acinetobacter baumannii* **(b)**, *Pectobacter wasabiae* **(c)**, and *Pseudomonas putida* **(d)** are shown. Exponentially growing cells were harvested by filtration and processed accordingly. *B. subtilis* sample was subjected to further refinement by FPLC and ethanol precipitation. Finally, samples were run on IPRP-HPLC-UV (black trace) and the identities of nucleotide peaks were revealed by a separate run with same samples but nucleotide standards spiked in. All cells were grown with vigorous aeration at 37 °C prior to harvesting. *B. subtilis* was grown in defined medium supplemented with glucose and amino acids (Kudrin, Varik et al. 2017), *P. wasabiae* was grown in defined medium with glucose (Koiv, Andresen et al. 2013), whereas both *A. baumannii* and *P. putida* were grown in LB.

HPLC conditions: (a-c) IPRP-HPLC, Kinetix C18 2.6 µm 4.6×150 mm, 26 °C. Buffer A: 5 mM TBA-OH, 30 mM KH_2PO_4 pH 6.0. Buffer B: 100% acetonitrile. Gradient: 0-20 min 5-35% B. (d) IPRP-HPLC, Symmetry C18 3.5 µm 4.6×150 mm; 0.8 ml/min, 26°C. A: 5 mM TBA-OH, 30 mM KH_2PO_4, pH 6.0; B: ACN. Gradient: 0-40 min 5-35% B.

SUPPLEMENTARY TABLES

Supplementary Table 1 | Cell concentration and dimensions. Cultures were started at OD_{600} 0.1 in defined minimal medium (MOPS 0.4% glucose at 37 °C with vigorous aeration). At 2, 4, 6, 8 and 10 hours of incubation, OD_{600} was determined, samples were taken for flow cytometry to count the number of cells, and for optical microscopy for determination of the dimensions. Dimensions were determined for 88-153 cells (n) for which mean and standard deviation (sd) were calculated. This table accompanies the **Supplementary Figure 7** to provide the numerical values.

			**Volume, fL**		**Length, µm**		**Width, µm**		
Time, h	OD_{600}	Cells/mL	Mean	sd	Mean	sd	Mean	sd	n
2	0.269	1.4×10^{8}	1.220	0.295	2.891	0.587	0.869	0.059	88
4	0.924	7.5×10^{8}	0.982	0.300	2.724	0.563	0.799	0.059	105
6	2.375	2.0×10^{9}	0.484	0.122	1.876	0.373	0.684	0.044	111
8	2.5	2.4×10^{9}	0.451	0.154	1.783	0.389	0.676	0.060	110
10	2.4	2.9×10^{9}	0.469	0.186	1.860	0.431	0.667	0.076	153

SUPPLEMENTARY REFERENCES

Arezzo, F. (1987). "Determination of ribonucleoside triphosphates and deoxyribonucleoside triphosphates in Novikoff hepatoma cells by high-performance liquid chromatography." Anal Biochem **160**(1): 57-64.

Atkinson, D. E. (1968). "The energy charge of the adenylate pool as a regulatory parameter. Interaction with feedback modifiers." Biochemistry **7**(11): 4030-4034.

Au, J. L., M. H. Su and M. G. Wientjes (1989). "Extraction of intracellular nucleosides and nucleotides with acetonitrile." Clin Chem **35**(1): 48-51.

Bagnara, A. S. and L. R. Finch (1968). "Ribonucleoside triphosphate accumulation on amino acid starvation of "stringent" Escherichia coli." Biochem Biophys Res Commun **33**(1): 15-21.

Bagnara, A. S. and L. R. Finch (1972). "Quantitative extraction and estimation of intracellular nucleoside triphosphates of Escherichia coli." Anal Biochem **45**(1): 24-34.

Bajad, S. U., W. Lu, E. H. Kimball, J. Yuan, C. Peterson and J. D. Rabinowitz (2006). "Separation and quantitation of water soluble cellular metabolites by hydrophilic interaction chromatography-tandem mass spectrometry." J Chromatogr A **1125**(1): 76-88.

Ball, W. J., Jr. and D. E. Atkinson (1975). "Adenylate energy charge in Saccharomyces cerevisiae during starvation." J Bacteriol **121**(3): 975-982.

Bennett, B. D., E. H. Kimball, M. Gao, R. Osterhout, S. J. Van Dien and J. D. Rabinowitz (2009). "Absolute metabolite concentrations and implied enzyme active site occupancy in Escherichia coli." Nat Chem Biol **5**(8): 593-599.

Bennett, B. D., J. Yuan, E. H. Kimball and J. D. Rabinowitz (2008). "Absolute quantitation of intracellular metabolite concentrations by an isotope ratio-based approach." Nat Protoc **3**(8): 1299-1311.

Bhattacharya, M., L. Fuhrman, A. Ingram, K. W. Nickerson and T. Conway (1995). "Single-run separation and detection of multiple metabolic intermediates by anion-exchange high-performance liquid chromatography and application to cell pool extracts prepared from Escherichia coli." Anal Biochem **232**(1): 98-106.

Bochner, B. R. and B. N. Ames (1982). "Complete analysis of cellular nucleotides by two-dimensional thin layer chromatography." J Biol Chem **257**(16): 9759-9769.

Boel, G., P. C. Smith, W. Ning, M. T. Englander, B. Chen, Y. Hashem, A. J. Testa, J. J. Fischer, H. J. Wieden, J. Frank, R. L. Gonzalez, Jr. and J. F. Hunt (2014). "The ABC-F protein EttA gates ribosome entry into the translation elongation cycle." Nat Struct Mol Biol **21**(2): 143-151.

Bolten, C. J., P. Kiefer, F. Letisse, J. C. Portais and C. Wittmann (2007). "Sampling for metabolome analysis of microorganisms." Anal Chem **79**(10): 3843-3849.

Brauer, M. J., J. Yuan, B. D. Bennett, W. Lu, E. Kimball, D. Botstein and J. D. Rabinowitz (2006). "Conservation of the metabolomic response to starvation across two divergent microbes." Proc Natl Acad Sci U S A **103**(51): 19302-19307.

Buchholz, A., J. Hurlebaus, C. Wandrey and R. Takors (2002). "Metabolomics: quantification of intracellular metabolite dynamics." Biomol Eng **19**(1): 5-15.

Buchholz, A., R. Takors and C. Wandrey (2001). "Quantification of intracellular metabolites in Escherichia coli K12 using liquid chromatographic-electrospray ionization tandem mass spectrometric techniques." Anal Biochem **295**(2): 129-137.

Buckstein, M. H., J. He and H. Rubin (2008). "Characterization of nucleotide pools as a function of physiological state in Escherichia coli." J Bacteriol **190**(2): 718-726.

Cabib, E., L. F. Leloir and C. E. Cardini (1953). "Uridine diphosphate acetylglucosamine." Journal of Biological Chemistry **203**(2): 1055-1070.

Cashel, M. (1969). "The control of ribonucleic acid synthesis in Escherichia coli. IV. Relevance of unusual phosphorylated compounds from amino acid-starved stringent strains." J Biol Chem **244**(12): 3133-3141.

Cashel, M. and J. Gallant (1968). "Control of RNA synthesis in Escherichia coli. I. Amino acid dependence of the synthesis of the substrates of RNA polymerase." J Mol Biol **34**(2): 317-330.

Cashel, M. and J. Gallant (1969). "Two compounds implicated in the function of the RC gene of Escherichia coli." Nature **221**(5183): 838-841.

Cashel, M. and B. Kalbacher (1970). "The control of ribonucleic acid synthesis in Escherichia coli. V. Characterization of a nucleotide associated with the stringent response." J Biol Chem **245**(9): 2309-2318.

Chapman, A. G., L. Fall and D. E. Atkinson (1971). "Adenylate energy charge in Escherichia coli during growth and starvation." J Bacteriol **108**(3): 1072-1086.

Chassagnole, C., N. Noisommit-Rizzi, J. W. Schmid, K. Mauch and M. Reuss (2002). "Dynamic modeling of the central carbon metabolism of Escherichia coli." Biotechnol Bioeng **79**(1): 53-73.

Cohn, W. E. (1949). "The Separation of Purine and Pyrimidine Bases and of Nucleotides by Ion Exchange." Science **109**(2833): 377-378.

Cole, H. A., J. W. Wimpenny and D. E. Hughes (1967). "The ATP pool in Escherichia coli. I. Measurement of the pool using modified luciferase assay." Biochim Biophys Acta **143**(3): 445-453.
Coulier, L., R. Bas, S. Jespersen, E. Verheij, M. J. van der Werf and T. Hankemeier (2006). "Simultaneous quantitative analysis of metabolites using ion-pair liquid chromatography-electrospray ionization mass spectrometry." Anal Chem **78**(18): 6573-6582.
Cross, D. R., B. J. Miller and S. J. James (1993). "A simplified HPLC method for simultaneously quantifying ribonucleotides and deoxyribonucleotides in cell extracts or frozen tissues." Cell Prolif **26**(4): 327-336.
Cserjan-Puschmann, M., W. Kramer, E. Duerrschmid, G. Striedner and K. Bayer (1999). "Metabolic approaches for the optimisation of recombinant fermentation processes." Appl Microbiol Biotechnol **53**(1): 43-50.
Dennis, P. P., M. Ehrenberg and H. Bremer (2004). "Control of rRNA synthesis in Escherichia coli: a systems biology approach." Microbiol Mol Biol Rev **68**(4): 639-668.
Dhople, A. M. and J. H. Hanks (1973). "Quantitative extraction of adenosine triphosphate from cultivable and host-grown microbes: calculation of adenosine triphosphate pools." Appl Microbiol **26**(3): 399-403.
Di Pierro, D., B. Tavazzi, C. F. Perno, M. Bartolini, E. Balestra, R. Calio, B. Giardina and G. Lazzarino (1995). "An ion-pairing high-performance liquid chromatographic method for the direct simultaneous determination of nucleotides, deoxynucleotides, nicotinic coenzymes, oxypurines, nucleosides, and bases in perchloric acid cell extracts." Anal Biochem **231**(2): 407-412.
Dominguez, H., C. Rollin, A. Guyonvarch, J. L. Guerquin-Kern, M. Cocaign-Bousquet and N. D. Lindley (1998). "Carbon-flux distribution in the central metabolic pathways of Corynebacterium glutamicum during growth on fructose." Eur J Biochem **254**(1): 96-102.
Edlin, G. and P. Broda (1968). "Physiology and genetics of the "ribonucleic acid control" locus in escherichia coli." Bacteriol Rev **32**(3): 206-226.
Edlin, G. and J. Neuhard (1967). "Regulation of nucleoside triphosphate pools in Escherichia coli." J Mol Biol **24**(2): 225-230.
Edlin, G. and G. S. Stent (1969). "Nucleoside triphosphate pools and the regulation of RNA synthesis in E. coli." Proc Natl Acad Sci U S A **62**(2): 475-482.
Fischer, M., T. P. Zimmerman and S. A. Short (1982). "A rapid method for the determination of guanosine 5'-diphosphate-3'-diphosphate and guanosine 5'-triphosphate-3'-diphosphate by high-performance liquid chromatography." Anal Biochem **121**(1): 135-139.
Fiske, C. H. (1934). "The Nature of the Depressor Substance of the Blood." Proc Natl Acad Sci U S A **20**(1): 25-27.
Franzen, J. S. and S. B. Binkley (1961). "Comparison of the acid-soluble nucleotides in Escherichia coli at different growth rates." J Biol Chem **236**: 515-519.
Fry, J. C. (1990). Direct Methods and Biomass Estimation. Methods in Microbiology. **22:** 41-85.
Gallant, J. and M. Cashel (1967). "On the mechanism of amino acid control of ribonucleic acid biosynthesis." J Mol Biol **25**(3): 545-553.
Gallant, J., H. Erlich, B. Hall and T. Laffler (1970). "Analysis of the RC Function." Cold Spring Harbor Symposia on Quantitative Biology **35**: 397-405.
Gallant, J. and B. Harada (1969). "The control of ribonucleic acid synthesis in Escherichia coli. 3. The functional relationship between purine ribonucleoside triphosphate pool sizes and the rate of ribonucleic acid accumulation." J Biol Chem **244**(12): 3125-3132.
Gallant, J., J. Irr and M. Cashel (1971). "The mechanism of amino acid control of guanylate and adenylate biosynthesis." J Biol Chem **246**(18): 5812-5816.
Garrett, C. and D. V. Santi (1979). "A rapid and sensitive high pressure liquid chromatography assay for deoxyribonucleoside triphosphates in cell extracts." Anal Biochem **99**(2): 268-273.
Gebelein, M., G. Merdes and M. R. Berger (1992). "Nucleotide preparation from cells and determination of nucleotides by ion-pair high-performance liquid chromatography." J Chromatogr **577**(1): 146-150.
Harmenberg, J., S. Cox and A. Akesson-Johansson (1990). "Improved sample preparation method for high-performance liquid chromatography of deoxyribonucleoside triphosphates from cell culture extracts." J Chromatogr **508**(1): 75-79.
Hiller, J., E. Franco-Lara and D. Weuster-Botz (2007). "Metabolic profiling of Escherichia coli cultivations: evaluation of extraction and metabolite analysis procedures." Biotechnol Lett **29**(8): 1169-1178.
Hoffman, N. E. and J. C. Liao (1977). "Reversed phase high performance liquid chromatographic separations of nucleotides in the presence of solvophobic ions." Anal Chem **49**(14): 2231-2234.
Holms, W. H., I. D. Hamilton and A. G. Robertson (1972). "The rate of turnover of the adenosine triphosphate pool of Escherichia coli growing aerobically in simple defined media." Arch Mikrobiol **83**(2): 95-109.
Irr, J. and J. Gallant (1969). "The control of ribonucleic acid synthesis in Escherichia coli. II. Stringent control of energy metabolism." J Biol Chem **244**(8): 2233-2239.
Ishii, N., K. Nakahigashi, T. Baba, M. Robert, T. Soga, A. Kanai, T. Hirasawa, M. Naba, K. Hirai, A. Hoque, P. Y. Ho, Y. Kakazu, K. Sugawara, S. Igarashi, S. Harada, T. Masuda, N. Sugiyama, T. Togashi, M. Hasegawa, Y.

Takai, K. Yugi, K. Arakawa, N. Iwata, Y. Toya, Y. Nakayama, T. Nishioka, K. Shimizu, H. Mori and M. Tomita (2007). "Multiple high-throughput analyses monitor the response of E. coli to perturbations." Science **316**(5824): 593-597.
Kaplan, N. O., S. P. Colowick and C. C. Barnes (1951). "Effect of alkali on diphosphopyridine nucleotide." J Biol Chem **191**(2): 461-472.
Khym, J. X. (1975). "An analytical system for rapid separation of tissue nucleotides at low pressures on conventional anion exchangers." Clin Chem **21**(9): 1245-1252.
Koebmann, B. J., H. V. Westerhoff, J. L. Snoep, D. Nilsson and P. R. Jensen (2002). "The glycolytic flux in Escherichia coli is controlled by the demand for ATP." J Bacteriol **184**(14): 3909-3916.
Koiv, V., L. Andresen, M. Broberg, J. Frolova, P. Somervuo, P. Auvinen, M. Pirhonen, T. Tenson and A. Mae (2013). "Lack of RsmA-mediated control results in constant hypervirulence, cell elongation, and hyperflagellation in Pectobacterium wasabiae." PLoS One **8**(1): e54248.
Kudrin, P., V. Varik, S. R. Oliveira, J. Beljantseva, T. Del Peso Santos, I. Dzhygyr, D. Rejman, F. Cava, T. Tenson and V. Hauryliuk (2017). "Subinhibitory Concentrations of Bacteriostatic Antibiotics Induce relA-Dependent and relA-Independent Tolerance to beta-Lactams." Antimicrob Agents Chemother **61**(4).
Lagosky, P. A. and F. N. Chang (1978). "The extraction of guanosine 5'-diphosphate, 3'-diphosphate (ppGpp) from Escherichia coli using low pH reagents: a reevaluation." Biochem Biophys Res Commun **84**(4): 1016-1024.
Lindberg, U. and L. Skoog (1970). "A method for the determination of dATP and dTTP in picomole amounts." Anal Biochem **34**: 152-160.
Little, R. and H. Bremer (1982). "Quantitation of guanosine 5',3'-bisdiphosphate in extracts from bacterial cells by ion-pair reverse-phase high-performance liquid chromatography." Anal Biochem **126**(2): 381-388.
Lowry, O. H., J. Carter, J. B. Ward and L. Glaser (1971). "The effect of carbon and nitrogen sources on the level of metabolic intermediates in Escherichia coli." J Biol Chem **246**(21): 6511-6521.
Lowry, O. H., J. V. Passonneau and M. K. Rock (1961). "The stability of pyridine nucleotides." J Biol Chem **236**: 2756-2759.
Lundin, A. and A. Thore (1975). "Comparison of methods for extraction of bacterial adenine nucleotides determined by firefly assay." Appl Microbiol **30**(5): 713-721.
Lundquist, R. and B. M. Olivera (1971). "Pyridine nucleotide metabolism in Escherichia coli. I. Exponential growth." J Biol Chem **246**(4): 1107-1116.
Luo, B., K. Groenke, R. Takors, C. Wandrey and M. Oldiges (2007). "Simultaneous determination of multiple intracellular metabolites in glycolysis, pentose phosphate pathway and tricarboxylic acid cycle by liquid chromatography-mass spectrometry." J Chromatogr A **1147**(2): 153-164.
Mack, D., V. Reed and L. Smith (1985). "Effect of pH, Temperature, and Ionic Strength on Reversed-Phase Ion-Pair High Performance Liquid Chroma-Tography of Purine Nucleotide Monophosphate." Journal of liquid chromatography **8**(4): 591-602.
Markham, R. and J. D. Smith (1952). "The structure of ribonucleic acid. I. Cyclic nucleotides produced by ribonuclease and by alkaline hydrolysis." Biochem J **52**(4): 552-557.
Markuszewski, M. J., P. Britz-McKibbin, S. Terabe, K. Matsuda and T. Nishioka (2003). "Determination of pyridine and adenine nucleotide metabolites in Bacillus subtilis cell extract by sweeping borate complexation capillary electrophoresis." J Chromatogr A **989**(2): 293-301.
Matin, A. and M. K. Matin (1982). "Cellular levels, excretion, and synthesis rates of cyclic AMP in Escherichia coli grown in continuous culture." J Bacteriol **149**(3): 801-807.
Meyer, H., M. Liebeke and M. Lalk (2010). "A protocol for the investigation of the intracellular Staphylococcus aureus metabolome." Anal Biochem **401**(2): 250-259.
Nazar, R. N., H. G. Lawford and J. T. Wong (1970). "An improved procedure for extraction and analysis of cellular nucleotides." Anal Biochem **35**(2): 305-313.
Neuhard, J. and A. Munch-Petersen (1966). "Studies on the acid-soluble nucleotide pool in thymine-requiring mutants of Escherichia coli during thymine starvation. II. Changes in the amounts of deoxycytidine triphosphate and deoxyadenosine triphosphate in Escherichia coli 15 T-A-U." Biochim Biophys Acta **114**(1): 61-71.
Nick McElhinny, S. A., B. E. Watts, D. Kumar, D. L. Watt, E. B. Lundstrom, P. M. Burgers, E. Johansson, A. Chabes and T. A. Kunkel (2010). "Abundant ribonucleotide incorporation into DNA by yeast replicative polymerases." Proc Natl Acad Sci U S A **107**(11): 4949-4954.
Ochi, K., J. C. Kandala and E. Freese (1981). "Initiation of Bacillus subtilis sporulation by the stringent response to partial amino acid deprivation." J Biol Chem **256**(13): 6866-6875.
Payne, S. H. and B. N. Ames (1982). "A procedure for rapid extraction and high-pressure liquid chromatographic separation of the nucleotides and other small molecules from bacterial cells." Anal Biochem **123**(1): 151-161.
Pogolotti, A. L., Jr. and D. V. Santi (1982). "High-pressure liquid chromatography--ultraviolet analysis of intracellular nucleotides." Anal Biochem **126**(2): 335-345.
Poulsen, P. and K. F. Jensen (1987). "Effect of UTP and GTP pools on attenuation at the pyrE gene of Escherichia coli." Mol Gen Genet **208**(1-2): 152-158.

Rabinowitz, J. D. (2007). "Cellular metabolomics of Escherchia coli." Expert Rev Proteomics **4**(2): 187-198.
Rabinowitz, J. D. and E. Kimball (2007). "Acidic acetonitrile for cellular metabolome extraction from Escherichia coli." Anal Chem **79**(16): 6167-6173.
Schaub, J., C. Schiesling, M. Reuss and M. Dauner (2006). "Integrated sampling procedure for metabolome analysis." Biotechnol Prog **22**(5): 1434-1442.
Schneider, D. A., T. Gaal and R. L. Gourse (2002). "NTP-sensing by rRNA promoters in Escherichia coli is direct." Proc Natl Acad Sci U S A **99**(13): 8602-8607.
Schneider, D. A. and R. L. Gourse (2004). "Relationship between growth rate and ATP concentration in Escherichia coli: a bioassay for available cellular ATP." J Biol Chem **279**(9): 8262-8268.
Schneider, D. A., H. D. Murray and R. L. Gourse (2003). "Measuring control of transcription initiation by changing concentrations of nucleotides and their derivatives." Methods Enzymol **370**: 606-617.
Skoog, L. (1970). "An enzymatic method for the determination of dCTP and dGTP in picomole amounts." Eur J Biochem **17**(2): 202-208.
Smith, R. C. and O. Maaloe (1964). "Effect of Growth Rate on the Acid-Soluble Nucleotide Composition of Salmonella Typhimurium." Biochim Biophys Acta **86**: 229-234.
Snyder, L. R., J. J. Kirkland and J. L. Glajch (1997). The Column. Practical HPLC Method Development, John Wiley & Sons, Inc.**:** 174-232.
Soga, T., Y. Ueno, H. Naraoka, K. Matsuda, M. Tomita and T. Nishioka (2002). "Pressure-assisted capillary electrophoresis electrospray ionization mass spectrometry for analysis of multivalent anions." Anal Chem **74**(24): 6224-6229.
Solter, A. W. and R. E. Handschumacher (1969). "A rapid quantitative determination of deoxyribonucleoside triphosphates based on the enzymatic synthesis of DNA." Biochim Biophys Acta **174**(2): 585-590.
Stock, J. B., B. Rauch and S. Roseman (1977). "Periplasmic space in Salmonella typhimurium and Escherichia coli." J Biol Chem **252**(21): 7850-7861.
Tanaka, K., A. Yoshioka, S. Tanaka and Y. Wataya (1984). "An improved method for the quantitative determination of deoxyribonucleoside triphosphates in cell extracts." Anal Biochem **139**(1): 35-41.
Taymaz-Nikerel, H., M. de Mey, C. Ras, A. ten Pierick, R. M. Seifar, J. C. van Dam, J. J. Heijnen and W. M. van Gulik (2009). "Development and application of a differential method for reliable metabolome analysis in Escherichia coli." Anal Biochem **386**(1): 9-19.
Vogel, U., S. Pedersen and K. F. Jensen (1991). "An unusual correlation between ppGpp pool size and rate of ribosome synthesis during partial pyrimidine starvation of Escherichia coli." J Bacteriol **173**(3): 1168-1174.
Walker-Simmons, M. and D. E. Atkinson (1977). "Functional capacities and the adenylate energy charge in Escherichia coli under conditions of nutritional stress." J Bacteriol **130**(2): 676-683.
Walsh, K. and D. E. Koshland, Jr. (1984). "Determination of flux through the branch point of two metabolic cycles. The tricarboxylic acid cycle and the glyoxylate shunt." J Biol Chem **259**(15): 9646-9654.
Wittmann, C., J. O. Kromer, P. Kiefer, T. Binz and E. Heinzle (2004). "Impact of the cold shock phenomenon on quantification of intracellular metabolites in bacteria." Anal Biochem **327**(1): 135-139.
